# Supplementary material for: Discovery of Anti-MRSA Secondary Metabolites from a Marine-Derived Fungus Aspergillus fumigatus
Source: Mar Drugs. 2022 Apr 28;20(5):302. doi: 10.3390/md20050302 (PMC9146929; doi:10.3390/md20050302)
Supplement: Supplementary file 1 [file marinedrugs-20-00302-s001.zip › marinedrugs-1699931-supplementary.pdf]

Supporting Information

Discovery of anti-MRSA secondary metabolites from a marine-derived fungus *Aspergillus fumigatus*

Rui Zhang [1,2], Haifeng Wang [1], Baosong Chen [2], Huanqin Dai [2], Jingzu Sun [2], Junjie Han [2*], and Hongwei Liu [1,2*]

[1] School of Traditional Chinese Materia Medica, Key Laboratory of Structure-Based Drug Design & Discovery of Education, Shenyang Pharmaceutical University, Shenyang 110016, China; zhangrui950714@163.com (R.Z.); wanghaifeng0310@163.com (H.W.); liuhw@im.ac.cn (H.L.).

[2] State Key Laboratory of Mycology, Institute of Microbiology, Chinese Academy of Sciences, Beijing 100101, China; hanjj@im.ac.cn (J.H.); chenbs@im.ac.cn (B.C.); daihq@im.ac.cn (H.D.); sunjz@im.ac.cn (J.S.); liuhw@im.ac.cn (H.L.).

* Correspondence: liuhw@im.ac.cn and hanjj@im.ac.cn; Tel.: +86-10-64806074.

Contents

Table S1. NMR calculation of **4**……………………………………………………...4

Table S2. sDP4+, uDP4+ and DP4+ probabilities (%) for **4**……………………………..6

Table S3. NMR calculation of **17**………………………………………………………….7

Table S4. sDP4+, uDP4+ and DP4+ probabilities (%) for **17**……………………………9

Figure S1. Phylogenetic tree of *A. fumigatus* H22……………………………………….10

Figure S2. Morphology of *A. fumigatus* H22…………………………………………….12

Figure S3. ^{1}H NMR spectrum of **1** (DMSO-d_6, 500 MHz)……………………………....13

Figure S4. ^{13}C NMR spectrum of **1** (DMSO-d_6, 125 MHz)……………………………...13

Figure S5. ^{1}H-^{1}H COSY spectrum of **1** (DMSO-d_6)……………………………………...14

Figure S6. HSQC spectrum of **1** (DMSO-d_6)……………………………………………..14

Figure S7. HMBC spectrum of **1** (DMSO-d_6)……………………………………………15

Figure S8. HRESIMS spectrum of **1**…………………………………………………....15

Figure S9. ^{1}H NMR spectrum of **2** (DMSO-d_6, 500 MHz)……………………………...16

Figure S10. ^{13}C NMR spectrum of **2** (DMSO-d_6, 125 MHz)……………………………16

Figure S11. ^{1}H-^{1}H COSY spectrum of **2** (DMSO-d_6)……………………………………17

Figure S12. HSQC spectrum of **2** (DMSO-d_6)……………………………………………...17

Figure S13. HMBC spectrum of **2** (DMSO-d_6)…………………………………………….18

Figure S14. ROESY spectrum of **2** (DMSO-d_6)…………………………………………....18

Figure S15. HRESIMS spectrum of **2**……………………………………………...19

Figure S16. ^{1}H NMR spectrum of **3** (DMSO-d_6, 500 MHz)……………………………19

Figure S17. ^{13}C NMR spectrum of **3** (DMSO-d_6, 125 MHz)…………………………...20

Figure S18. ^{1}H-^{1}H COSY spectrum of **3** (DMSO-d_6)…………………………………...20

Figure S19. HSQC spectrum of **3** (DMSO-d_6)……………………………………………..21

Figure S20. HMBC spectrum of **3** (DMSO-d_6)…………………………………………….21

Figure S21. ROESY spectrum of **3** (DMSO-d_6)……………………………………………22

Figure S22. HRESIMS spectrum of **3**…………………………………………………22

Figure S23. Scheme of the resonance structure of **3** and the chemical equilibrium between **3a** and **3b**...23

Figure S24. Key ^{1}H-^{1}H COSY, HMBC, and ROESY correlations of **3a** and **3b**..........23

Figure S25. ^{1}H NMR spectrum of **4** ($CDCl_3$-*d*, 500 MHz)…………………………...24

Figure S26. ^{13}C NMR spectrum of **4** ($CDCl_3$-*d*, 125 MHz)……………………………..24

Figure S27. ^{1}H-^{1}H COSY spectrum of **4** ($CDCl_3$-*d*)……………………………………..25

Figure S28. HSQC spectrum of **4** ($CDCl_3$-*d*)……………………………………….....25

Figure S29. HMBC spectrum of **4** ($CDCl_3$-*d*)……………………………………….....26

Figure S30. ROESY spectrum of **4** ($CDCl_3$-*d*)……………………………………...26

Figure S31. HRESIMS spectrum of **4**…………………………………………………27

Figure S32. ^{1}H NMR spectrum of **17** ($CDCl_3$-*d*, 500 MHz)……………………………27

Figure S33. ^{13}C NMR spectrum of **17** ($CDCl_3$-*d*, 125 MHz)……………………………28

Figure S34. HSQC spectrum of **17** ($CDCl_3$-*d*)……………………………………...28

Figure S35. HMBC spectrum of **17** ($CDCl_3$-*d*)……………………………………....29

Figure S36. ROESY spectrum of **17** ($CDCl_3$-*d*)……………………………………...29

Figure S37. HRESIMS spectrum of **17**…………………………………………………30

Figure S38. ^{1}H NMR spectrum of **37** (DMSO-d_6, 500 MHz)…………………………....30

Figure S39. ^{13}C NMR spectrum of **37** (DMSO-d_6, 125 MHz)…………………………...31

Figure S40. HSQC spectrum of **37** (DMSO-d_6)……………………………………..31

Figure S41. HMBC spectrum of **37** (DMSO-d_6)…………………………………….32

Figure S42. HRESIMS spectrum of **37**…………………………………………………32

Figure S43. Eight possible stereoisomers of **4** (**4a**–**4h**)…………………………………33

Figure S44. Four possible stereoisomers of **17** (**17a**–**17d**)………………………………33

Table S1. NMR calculation of **4**.

Experimental chemical shifts, and Boltzmann averaged GIAO isotropic magnetic shielding values calculated for mPW1PW91/6-31G* geometries of compounds **4** (Figure **S43**)

Level of theory: mPW1PW91/6-31G* (solution, PCM)

Type of Data (Shielding Tensors)

	Exp	**4a** (3*S*6*S*12*R*13*R*)	**4b** (3*S*6*R*12*S*13*R*)	**4c** (3*S*6*S*12*R*13*S*)	**4d** (3*S*6*R*12*S*13*S*)	**4e** (3*S*6*R*12*R*13*S*)	**4f** (3*S*6*R*12*R*13*R*)	**4g** 3*S*6*S*12*S*13*S*)	**4h** (3*S*6*S*12*S*13*R*)
^{13}C									
2	137.0	137.53	142.43	132.40	137.71	135.52	140.47	138.79	137.02
3	43.3	50.96	48.98	51.25	51.10	50.31	58.37	49.97	51.40
5	164.7	175.17	169.96	173.78	168.57	173.80	170.19	168.69	167.41
6	59.8	62.83	60.92	61.85	61.05	64.19	64.87	63.35	62.69
7	29.6	29.73	29.07	29.25	27.74	30.66	31.66	32.36	31.78
8	22.0	25.14	25.01	26.66	25.44	24.23	23.91	23.92	24.28
9	45.7	46.87	47.15	47.46	46.64	45.91	47.24	46.62	47.64
11	165.9	164.33	163.57	166.40	168.15	168.60	165.01	163.81	164.61
12	86.2	86.51	85.99	85.41	86.32	83.41	88.56	88.55	86.33
13	68.4	67.64	62.09	70.95	75.66	69.98	72.22	70.71	69.49
14	114.3	113.68	116.24	114.47	117.93	112.87	117.75	116.65	117.98
15	122.3	120.76	122.27	121.15	121.11	121.80	123.31	121.86	122.88
16	119.4	117.73	119.27	121.00	121.02	121.71	118.60	118.64	121.21
17	111.2	113.68	112.74	113.51	111.36	108.01	105.86	107.81	108.69
18	157.7	157.76	156.16	157.39	156.26	155.75	155.59	156.02	155.84
19	100.8	97.60	95.35	98.86	98.37	99.33	103.87	100.62	99.10
20	136.1	138.10	134.41	138.41	135.64	135.99	136.68	135.77	135.81
21	39.5	50.34	45.55	49.68	50.20	49.83	39.07	42.91	41.96
22	74.6	69.25	75.03	69.66	69.39	70.37	73.07	72.94	72.96
23	29.3	27.71	32.98	28.86	25.81	29.39	28.54	31.39	32.43

24	32.2	31.96	25.46	33.19	30.75	28.20	31.80	26.54	26.29
25	165.5	166.13	165.02	165.42	166.26	164.90	164.85	165.25	165.23
26	119.8	119.43	118.97	119.62	118.25	118.98	119.09	119.18	119.39
27	158.2	167.51	167.87	168.90	170.91	167.61	165.72	167.09	166.01
28	27.4	27.23	27.56	28.08	28.29	24.99	26.85	23.74	26.11
29	21.2	22.45	22.43	24.05	23.16	25.13	22.59	26.08	23.33
18-OCH_3	55.9	53.78	53.26	54.52	53.49	53.51	53.87	53.64	53.49
^1H									
3	6.37	6.04	6.05	6.04	5.82	6.00	5.38	6.40	6.18
6	4.32	4.57	4.41	4.61	4.54	3.98	4.38	4.27	4.11
7	2.51	2.47	2.40	2.97	2.70	1.89	2.25	1.97	1.95
	1.95	2.27	2.10	2.32	1.95	2.22	1.96	2.10	2.29
8	2.08	1.88	1.69	1.96	1.38	1.94	1.96	1.93	1.91
	1.98	1.84	1.76	1.85	1.62	2.02	1.99	1.93	1.94
9	3.76	3.35	3.51	3.26	3.64	3.81	3.61	3.46	3.45
	3.65	3.69	3.24	4.04	2.91	3.24	3.55	3.49	3.52
13	5.13	5.39	6.04	5.80	5.35	5.53	4.71	4.91	5.07
16	7.45	7.51	7.60	7.99	7.89	7.89	7.52	7.51	7.84
17	6.90	6.96	6.90	7.03	6.87	6.81	6.85	6.86	6.81
19	7.27	7.97	7.08	8.23	7.07	7.17	7.25	7.18	7.13
21	2.29	2.64	2.36	1.96	2.30	2.23	3.88	2.43	2.44
	2.14	1.79	3.12	2.09	1.87	1.65	2.74	2.64	2.52
23	1.25	0.98	1.22	1.17	1.63	1.03	0.40	1.17	1.15
24	1.17	1.09	1.72	1.31	1.09	1.56	1.17	1.38	1.32
26	6.40	6.44	6.51	6.80	6.44	6.48	6.43	6.47	6.46
28	2.11	2.27	2.16	2.49	2.13	2.23	2.12	2.27	2.18
29	2.21	2.24	2.34	2.40	2.36	2.25	2.26	2.18	2.24
18-OCH_3	3.85	3.78	3.72	3.92	3.73	3.75	3.77	3.75	3.73

Table S2. sDP4+, uDP4+ and DP4+ probabilities (%) for **4**

		Level of theory: mPW1PW91/6-31G* (solution, PCM)							
		**4a** (3*S*6*S*12*R*13*R*)	**4b** (3*S*6*R*12*S*13*R*)	**4c** (3*S*6*S*12*R*13*S*)	**4d** (3*S*6*R*12*S*13*S*)	**4e** (3*S*6*R*12*R*13*S*)	**4f** (3*S*6*R*12*R*13*R*)	**4g** (3*S*6*S*12*S*13*S*)	**4h** (3*S*6*S*12*S*13*R*)
sDP4+	^{1}H data	13.65	0.00	0.00	0.00	0.00	0.00	75.37	10.97
	^{13}C data	0.00	0.00	0.00	0.00	0.00	0.01	0.08	99.91
	All data	0.00	0.00	0.00	0.00	0.00	0.00	0.52	99.48
uDP4+	^{1}H data	0.00	0.00	0.00	0.00	0.00	0.00	95.63	4.37
	^{13}C data	0.00	0.00	0.00	0.00	0.00	7.27	41.32	51.25
	All data	0.00	0.00	0.00	0.00	0.00	0.00	94.64	5.36
DP4+	^{1}H data	0.00	0.00	0.00	0.00	0.00	0.00	99.34	0.66
	^{13}C data	0.00	0.00	0.00	0.00	0.00	0.00	0.06	99.94
	All data	0.00	0.00	0.00	0.00	0.00	0.00	8.45	91.55

Table S3. NMR calculation of **17**.

Experimental chemical shifts, and Boltzmann averaged GIAO isotropic magnetic shielding values calculated for mPW1PW91/6-31G* geometries of compounds **17** (Figure **S44**).

		Level of theory: mPW1PW91/6-31G* (solution, PCM)				
		Type of Data (Shielding Tensors)				
		Exp	**17a** (2*S*, 3*S*, 12***R***, 15*S*)	**17b** (2*S*, 3***R***, 12*S*, 15*S*)	**17c** (2*S*, 3***R***, 12***R***, 15*S*)	**17d** (2*S*, 3*S*, 12*S*, 15*S*)
^{13}C	2	82.6	85.1	86.9	85.7	84.6
	3	91.3	93.6	87.0	84.6	92.4
	4	134.4	135.5	134.0	132.3	136.3
	5	124.4	125.0	124.7	127.7	126.1
	6	125.8	123.5	123.7	123.8	125.1
	7	131.3	130.0	130.6	131.2	129.6
	8	115.8	114.5	115.6	113.7	115.1
	9	138.9	140.8	141.4	139.8	137.2
	11	170.9	171.9	170.8	170.4	171.9
	12	58.3	61.7	61.1	60.6	60.9
	13	33.4	36.7	38.6	38.2	35.4
	14	172.4	173.6	175.2	173.6	170.2
	15	58.5	59.3	60.3	63.4	61.8
	18	161.0	160.4	160.0	159.5	159.5
	19	121.9	121.8	121.5	122.0	122.1
	20	126.8	126.3	126.8	127.1	127.1
	21	128.2	126.7	126.9	126.5	126.5
	22	135.4	134.4	134.5	134.3	134.0
	23	128.0	128.3	128.3	128.0	127.9
	24	148.1	148.2	148.1	148.1	147.9

	26	145.6	147.4	147.3	147.4	147.6
	27	17.9	17.7	17.9	14.8	19.6
^{1}H	2	5.82	5.61	5.25	5.12	5.48
	5	7.31	7.37	7.43	8.21	8.03
	6	7.22	7.19	7.22	7.27	7.33
	7	7.43	7.41	7.45	7.48	7.38
	8	7.65	7.70	7.68	7.61	7.48
	12	5.02	4.64	4.71	4.69	4.52
	13	3.68	3.71	2.82	3.67	2.65
		2.61	2.59	3.12	2.76	3.37
	15	4.26	4.11	4.06	3.84	4.21
	20	8.29	8.26	8.34	8.39	8.36
	21	7.58	7.55	7.57	7.57	7.54
	22	7.85	7.82	7.83	7.78	7.77
	23	7.78	7.73	7.74	7.72	7.68
	26	8.11	7.75	7.75	7.71	7.65
	27	1.28	1.19	1.41	1.37	1.37

Table S4. sDP4+, uDP4+ and DP4+ probabilities (%) for **17**.

		Level of theory: mPW1PW91/6-31G* (solution, PCM)			
		**17a** (2*S*, 3*S*, 12***R***, 15*S*)	**17b** (2*S*, 3***R***, 12*S*, 15*S*)	**17c** (2*S*, 3***R***, 12***R***, 15*S*)	**17d** (2*S*, 3*S*, 12*S*, 15*S*)
sDP4+	^{1}H data	100.00	0.00	0.00	0.00
	^{13}C data	4.72	0.03	0.00	95.25
	All data	100.00	0.00	0.00	0.00
uDP4+	^{1}H data	99.67	0.33	0.00	0.00
	^{13}C data	93.44	0.29	0.00	6.27
	All data	100.00	0.00	0.00	0.00
DP4+	^{1}H data	100.00	0.01	0.00	0.00
	^{13}C data	42.48	0.00	0.00	57.52
	All data	100.00	0.00	0.00	0.00

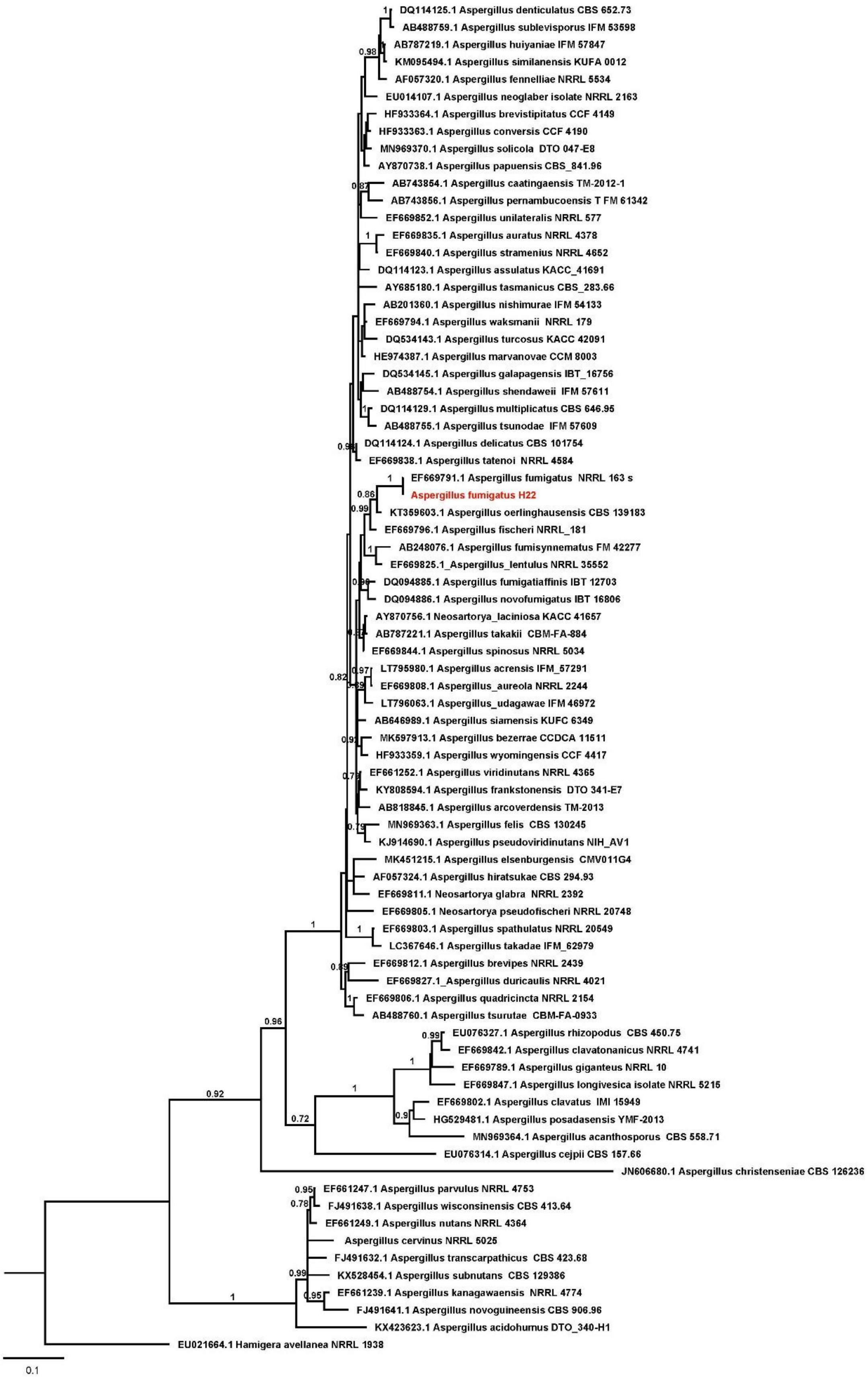

Figure S1. Phylogenetic tree of *A. fumigatus* H22. Neighbor-joining tree of *A.*

fumigatus H22 based on 18S sequences. Numbers at nodes indicate levels of bootstrap support (%) based on a neighbor-joining analysis of 1000 resampled datasets; only values >50 % are shown. National Center for Biotechnology Information (NCBI) accession numbers are provided in parentheses. The Bar represents 0.05 nucleotide substitutions per site.

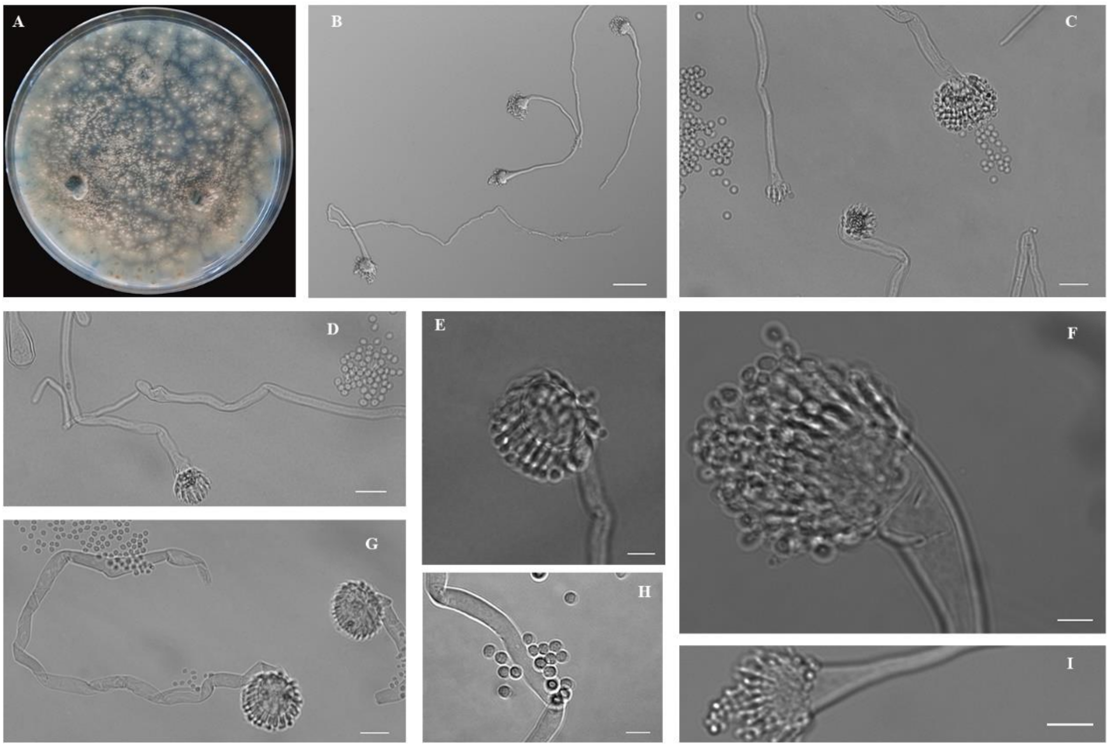

Figure S2. Morphology of *A. fumigatus* H22. Colony characteristics of *A. fumigatus* H22 grown on potato dextrose agar at 28 ℃ for 7 days. A on PDA; B, E, H, F, I conidia and phialides (Scale bars: 20 μm); C, D, G conidia and phialides (Scale bars: 20 μm).

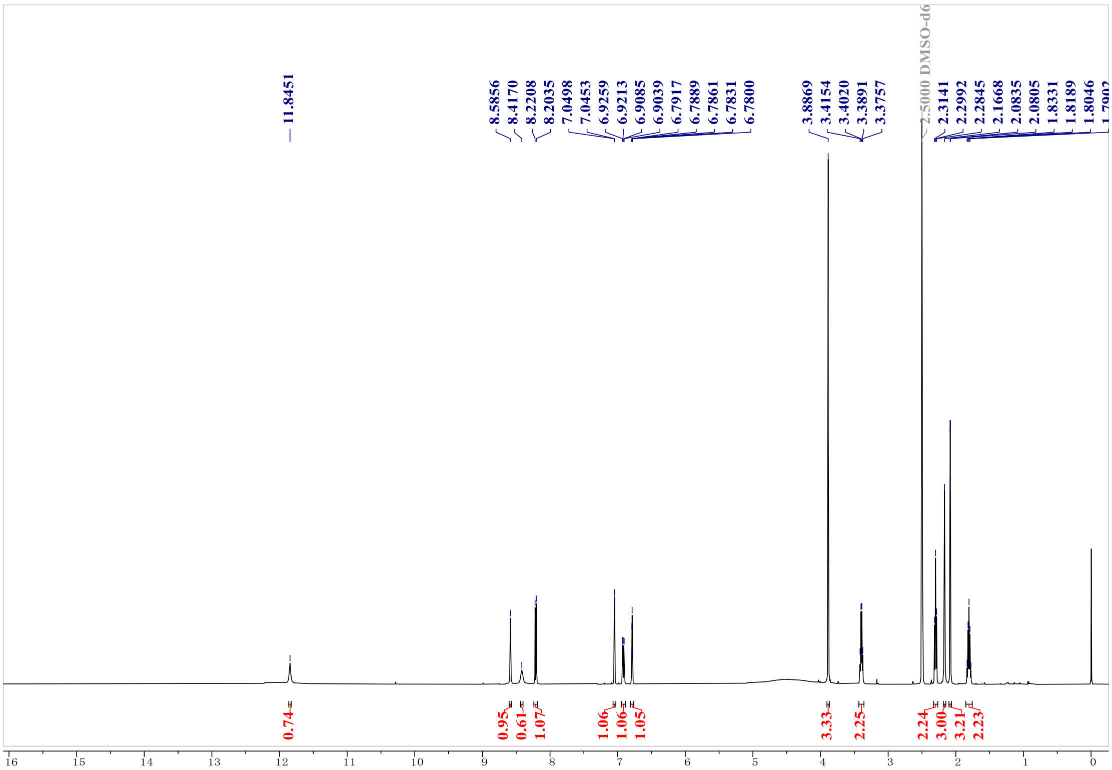

Figure S3. ^{1}H NMR spectrum of **1** (DMSO-*d*$_6$, 500 MHz).

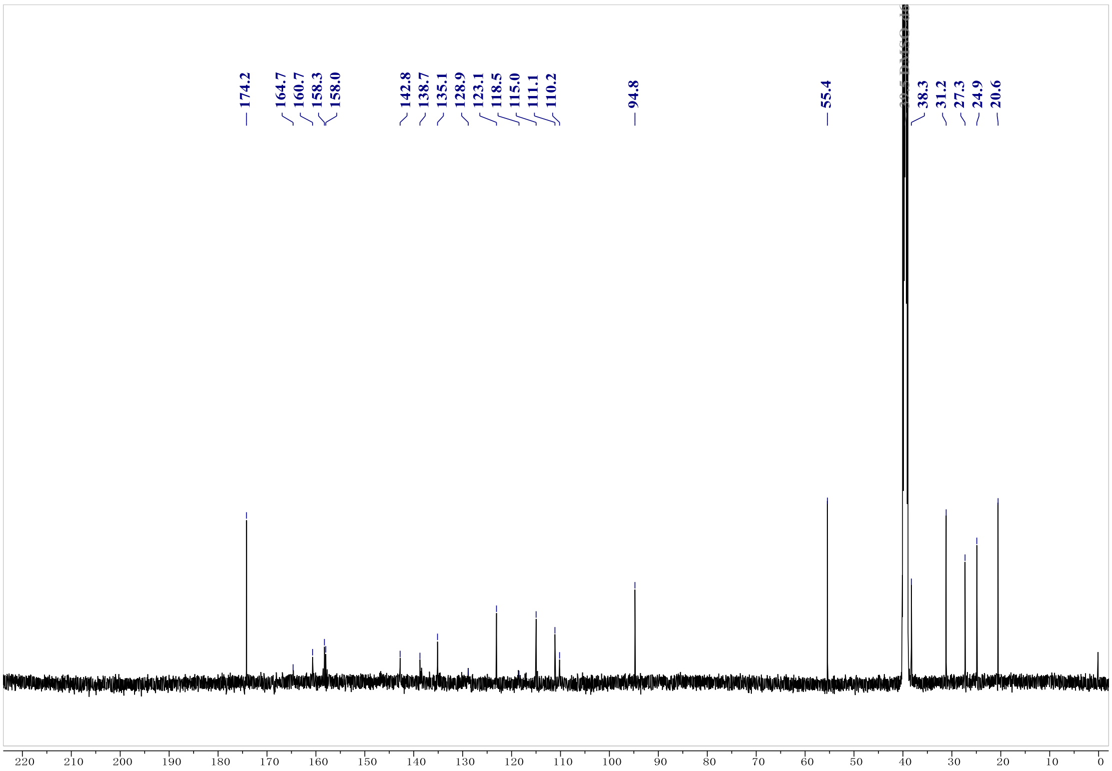

Figure S4. ^{13}C NMR spectrum of **1** (DMSO-*d*$_6$, 125 MHz).

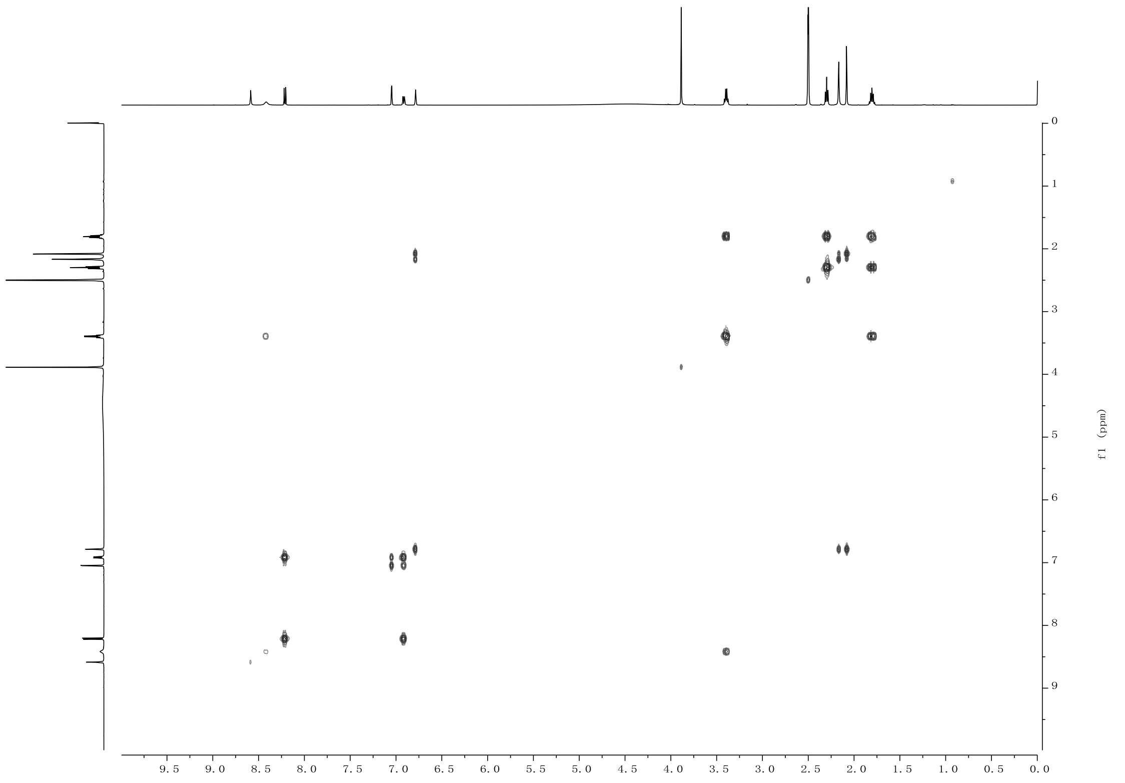

Figure S5. ^{1}H-^{1}H COSY spectrum of **1** (DMSO-d_6).

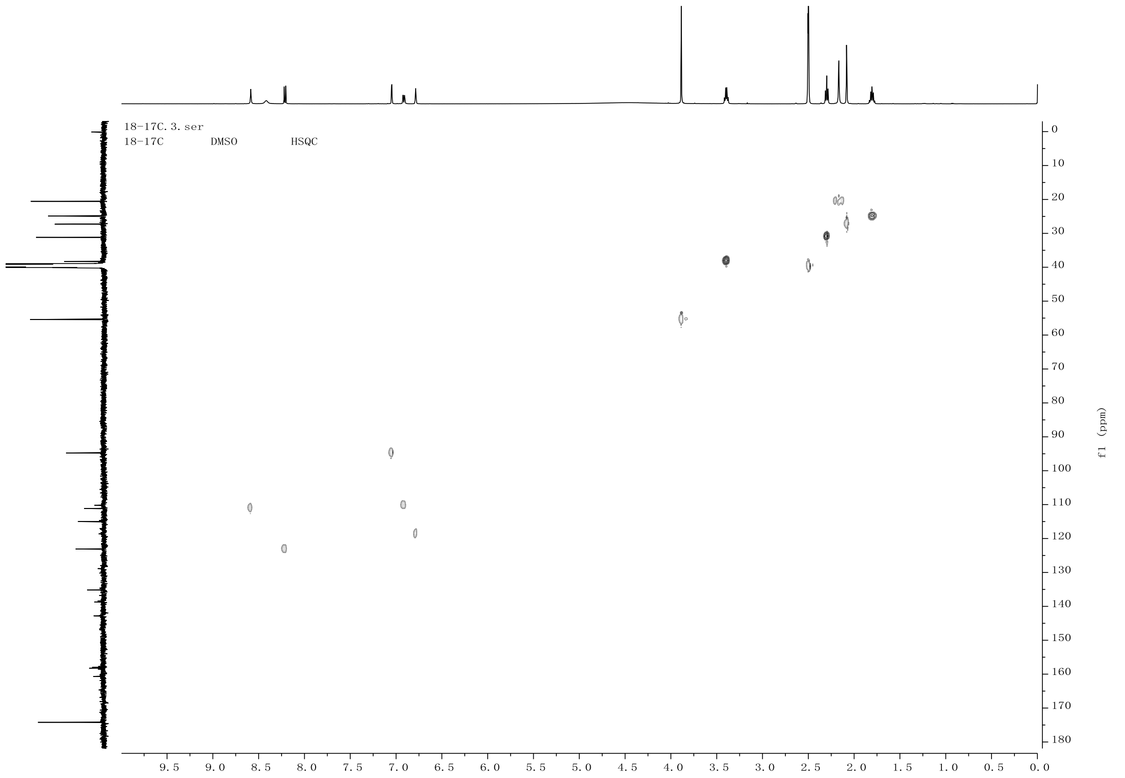

Figure S6. HSQC spectrum of **1** (DMSO-d_6).

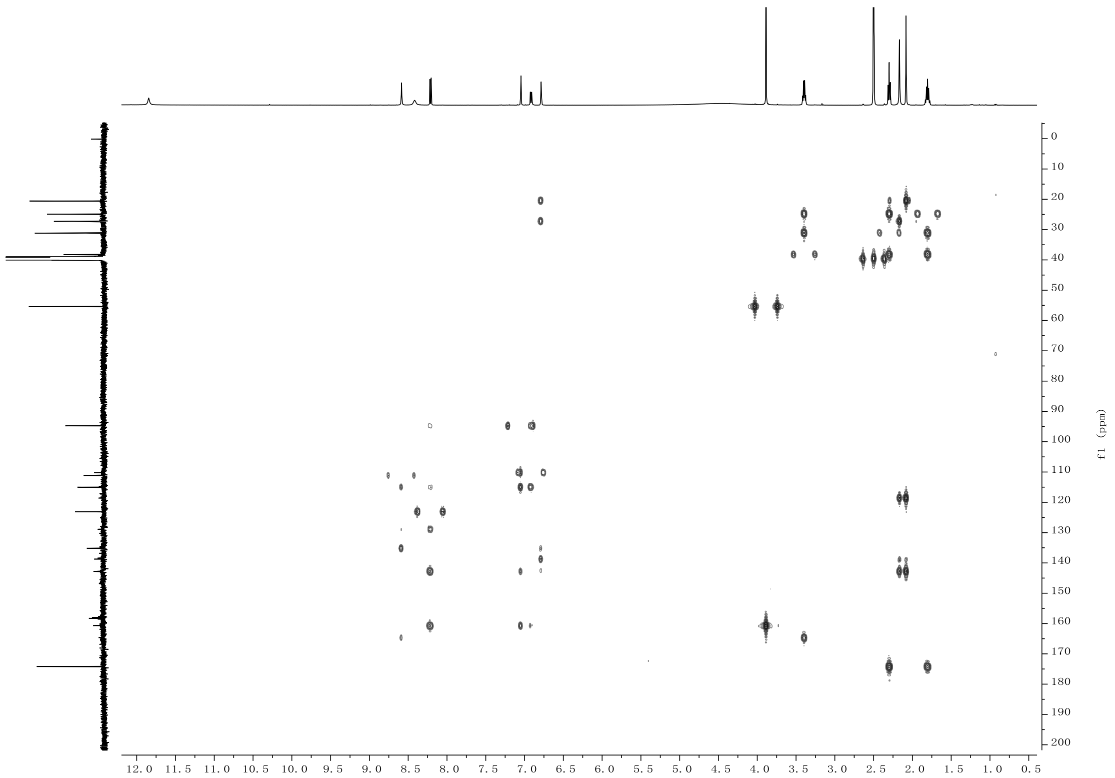

Figure S7. HMBC spectrum of **1** (DMSO-d_6).

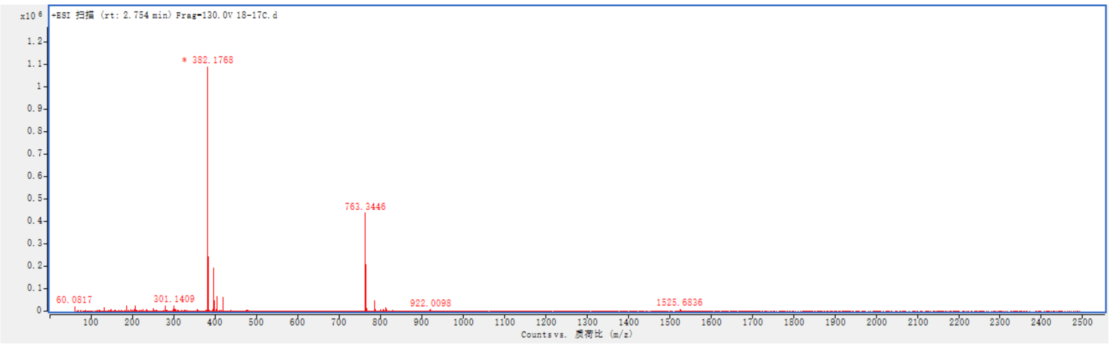

Figure S8. HRESIMS spectrum of **1**.

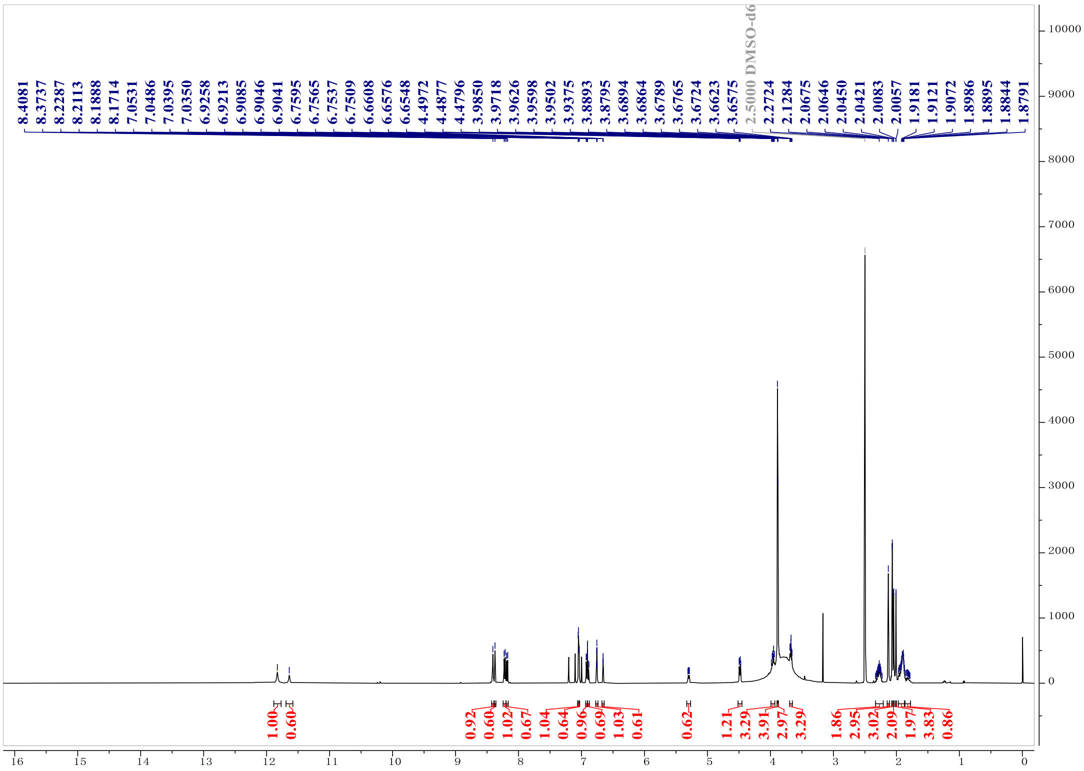

Figure S9. ^{1}H NMR spectrum of **2** (DMSO-d_6, 500 MHz).

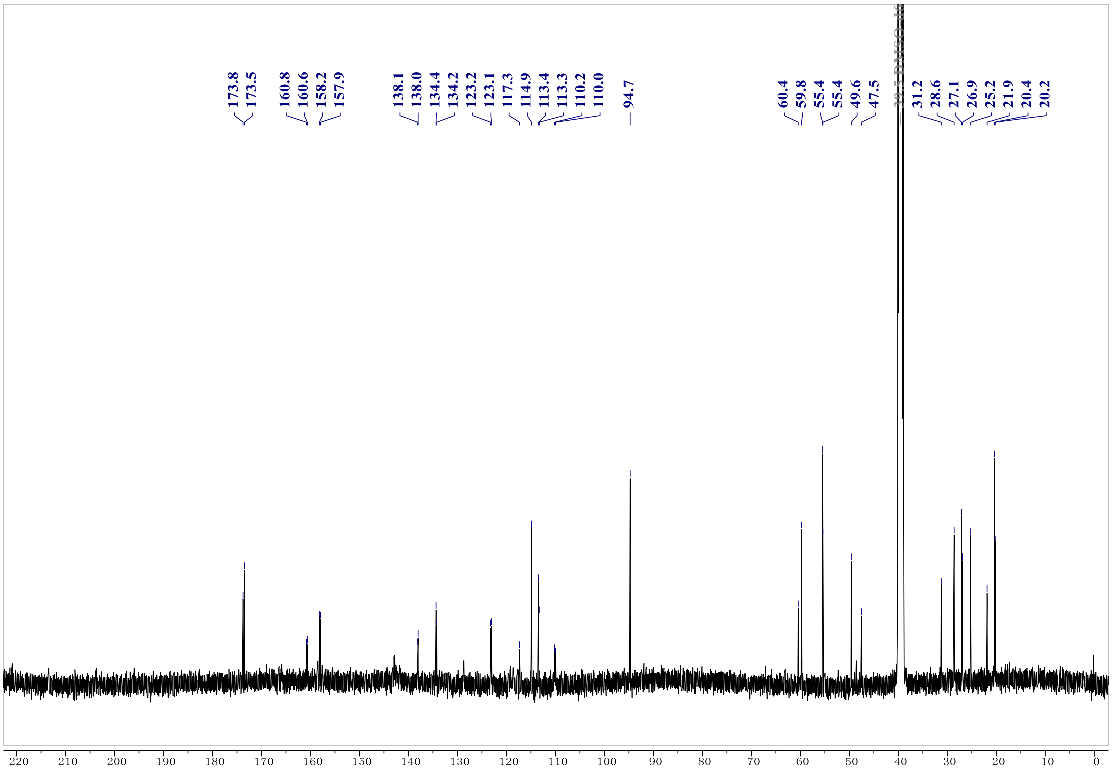

Figure S10. ^{13}C NMR spectrum of **2** (DMSO-d_6, 125 MHz).

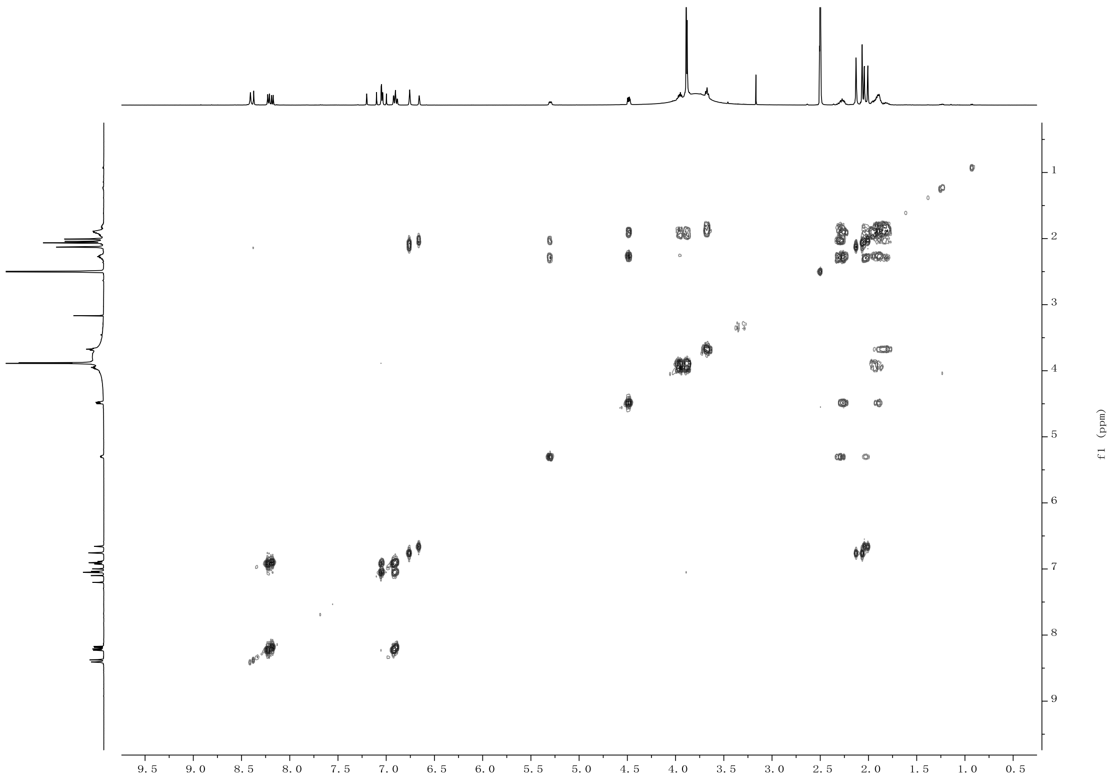

Figure S11. ^1H-^1H COSY spectrum of **2** (DMSO-d_6).

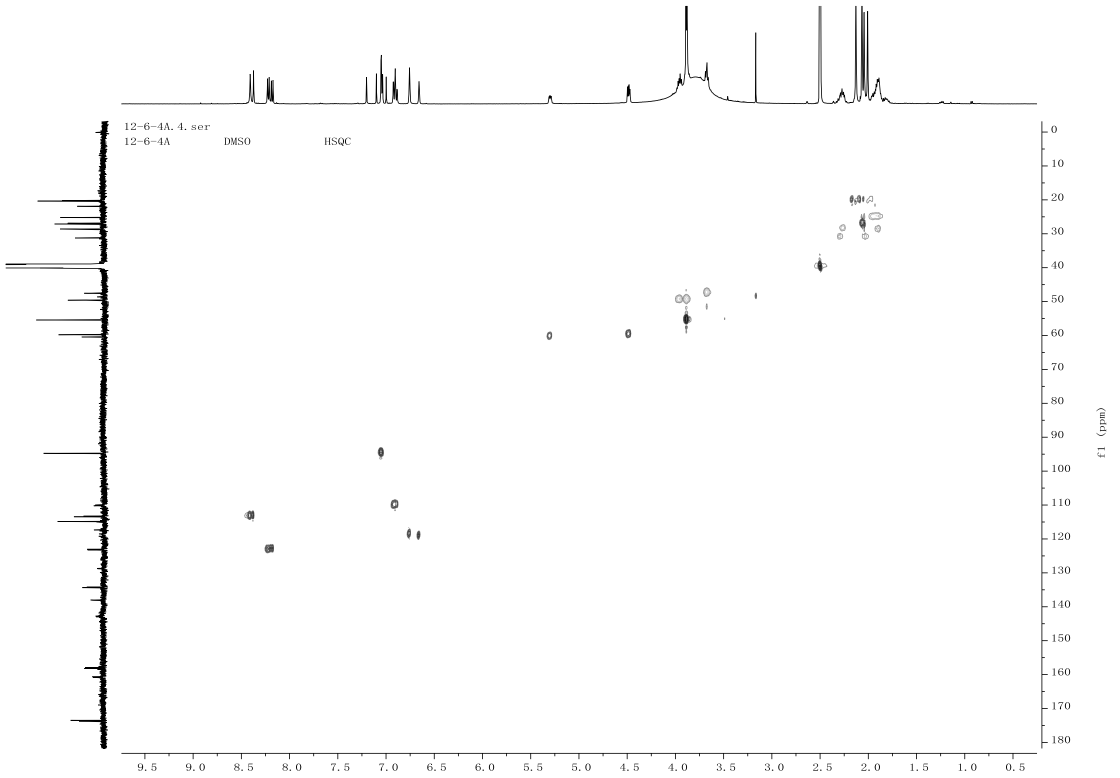

Figure S12. HSQC spectrum of **2** (DMSO-d_6).

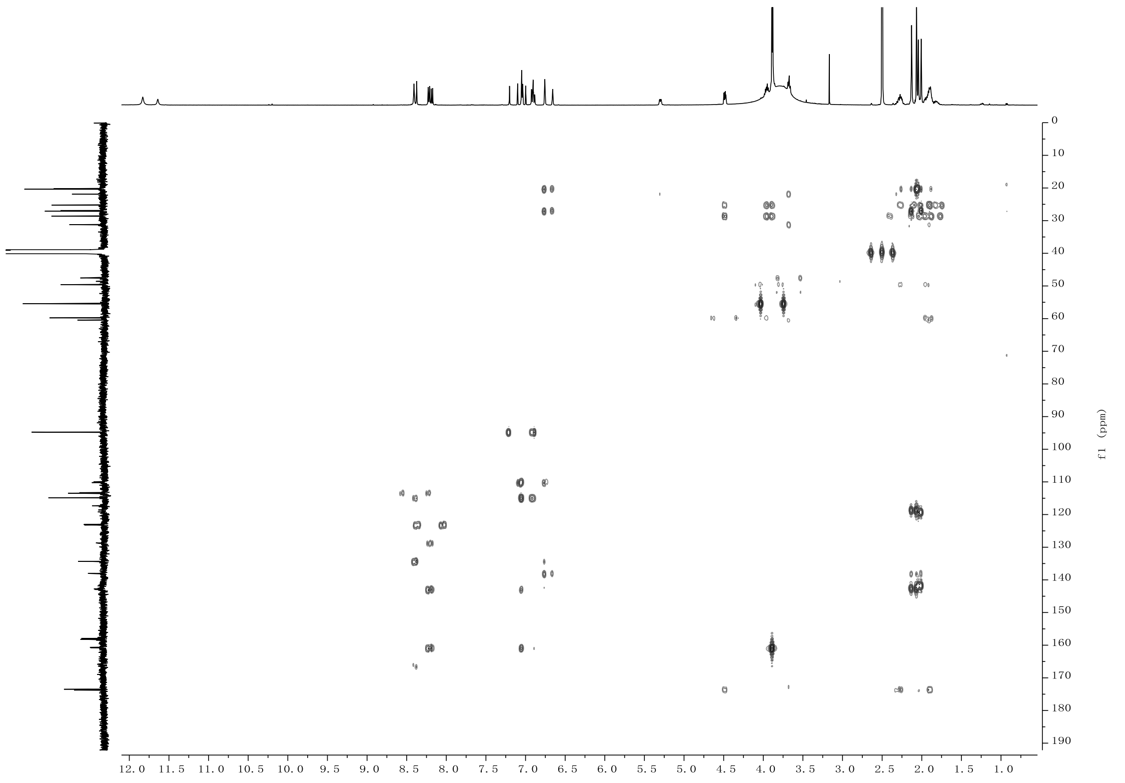

Figure S13. HMBC spectrum of **2** (DMSO-d_6).

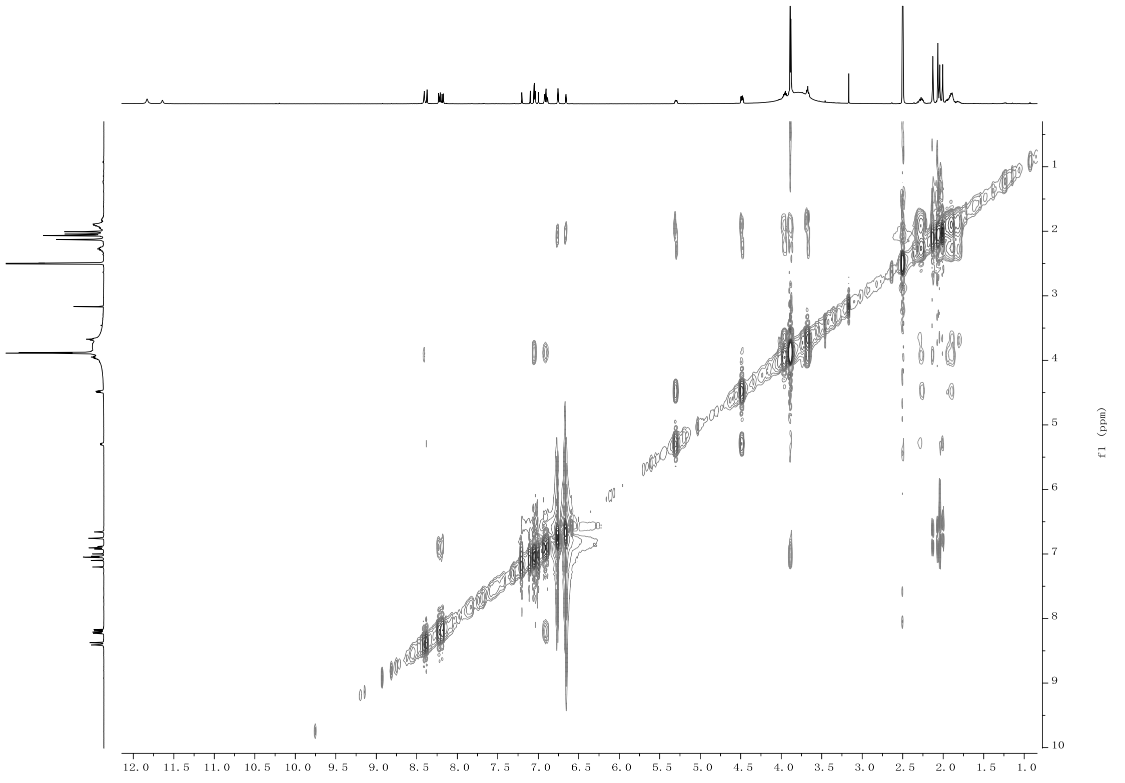

Figure S14. ROESY spectrum of **2** (DMSO-d_6).

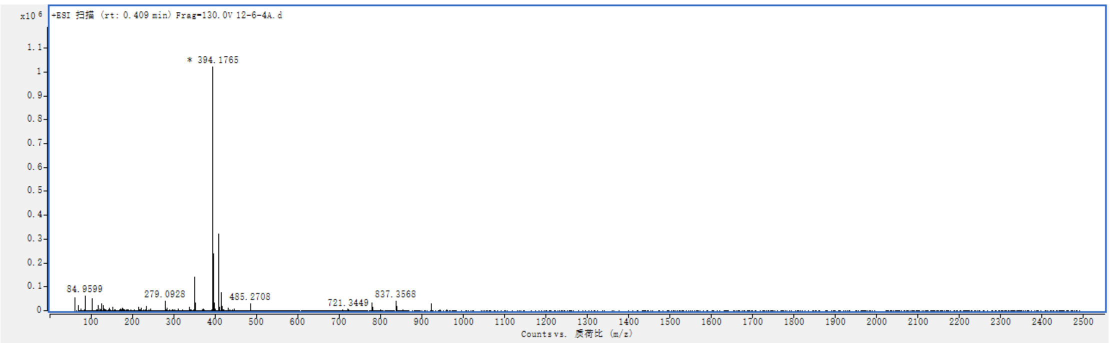

Figure S15. HRESIMS spectrum of **2**.

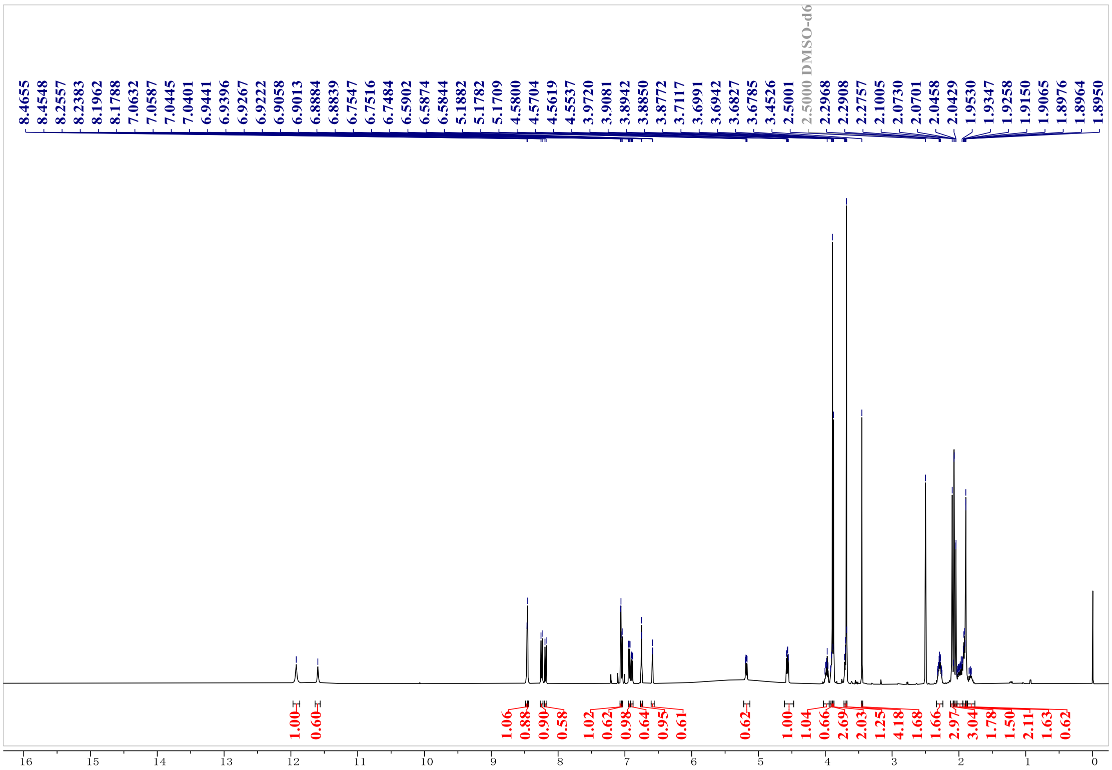

Figure S16. ^{1}H NMR spectrum of **3** (DMSO-d_6, 500 MHz).

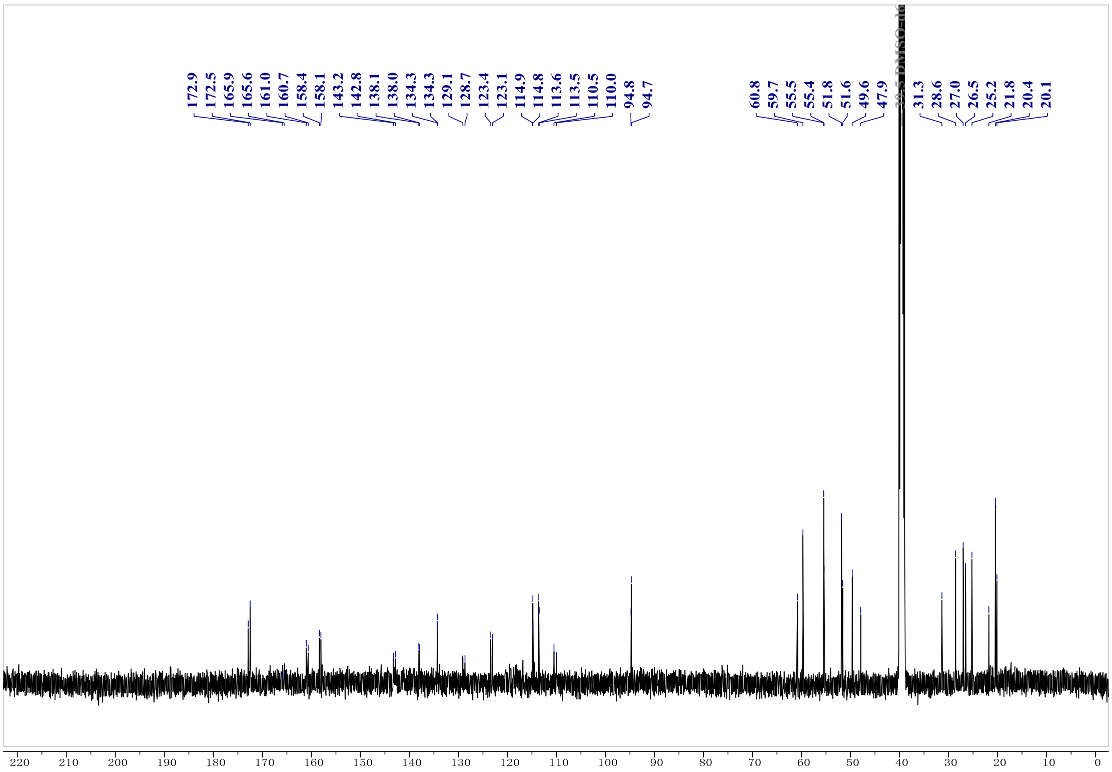

Figure S17. ^{13}C NMR spectrum of **3** (DMSO-d_6, 125 MHz).

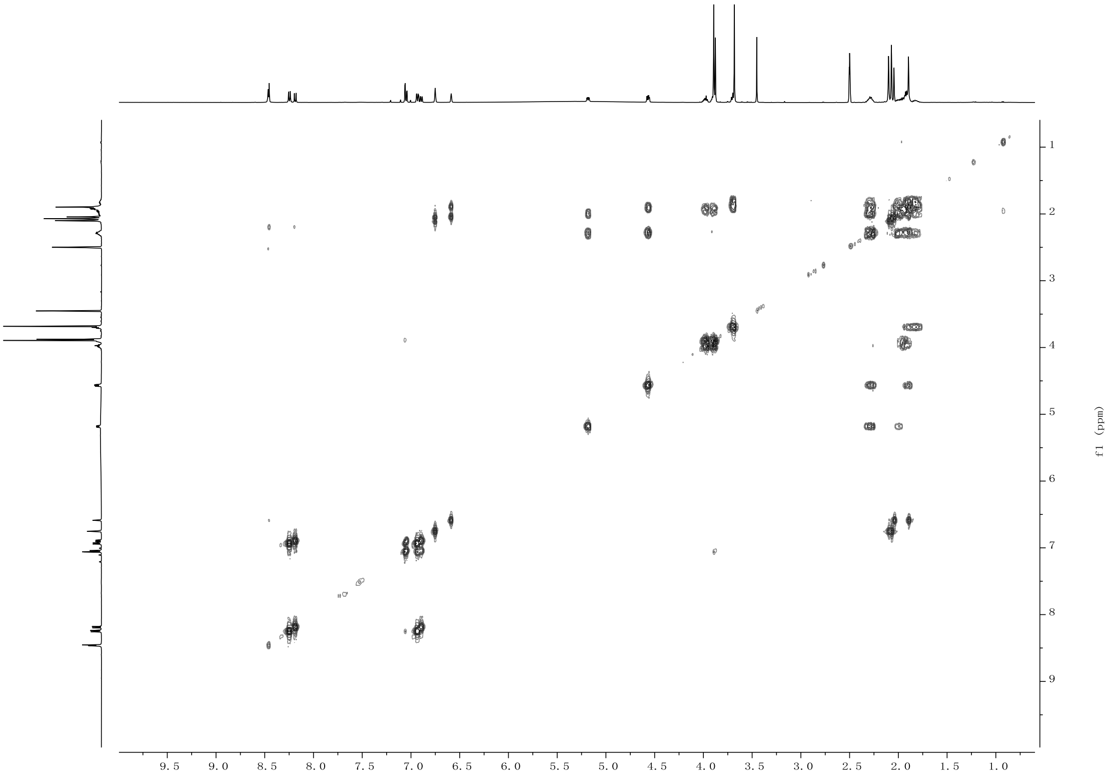

Figure S18. ^{1}H-^{1}H COSY spectrum of **3** (DMSO-d_6).

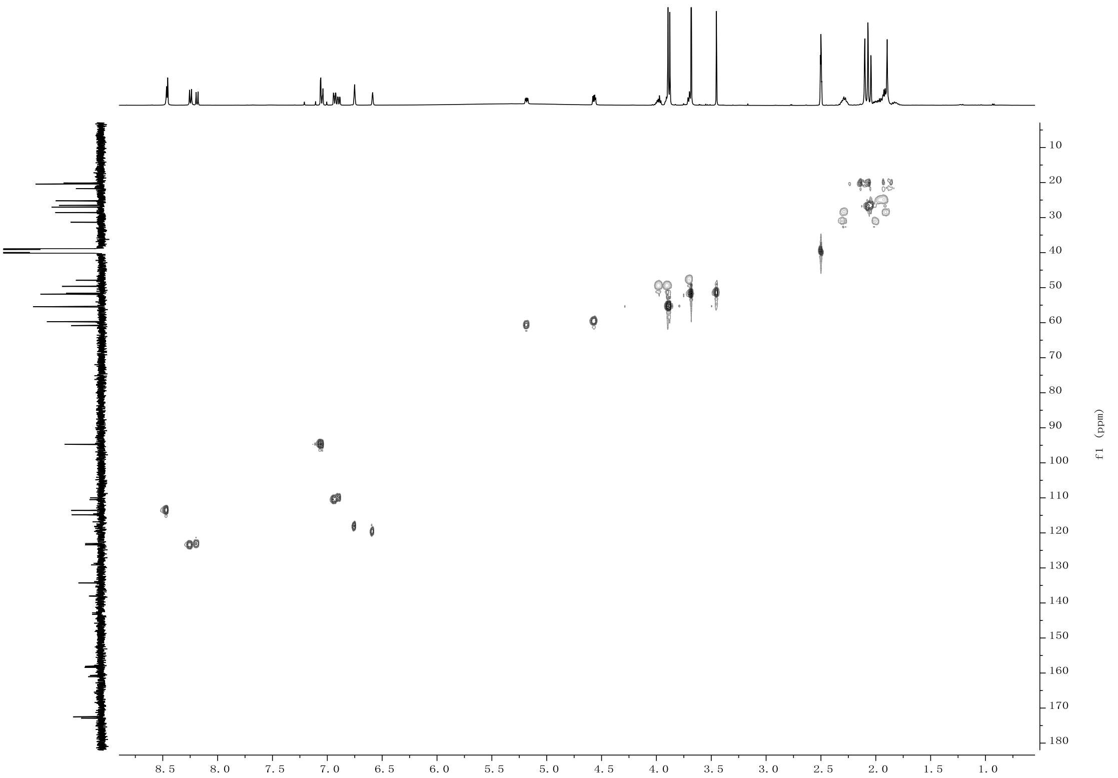

Figure S19. HSQC spectrum of **3** (DMSO-*d*6).

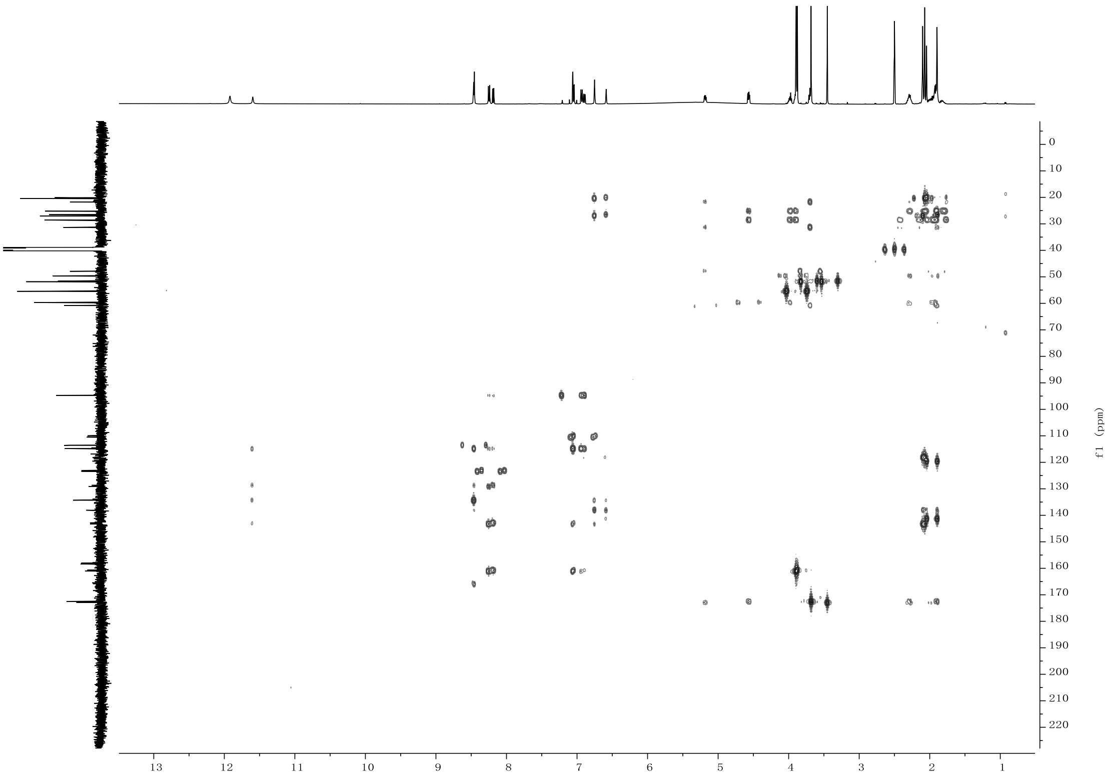

Figure S20. HMBC spectrum of **3** (DMSO-*d*6).

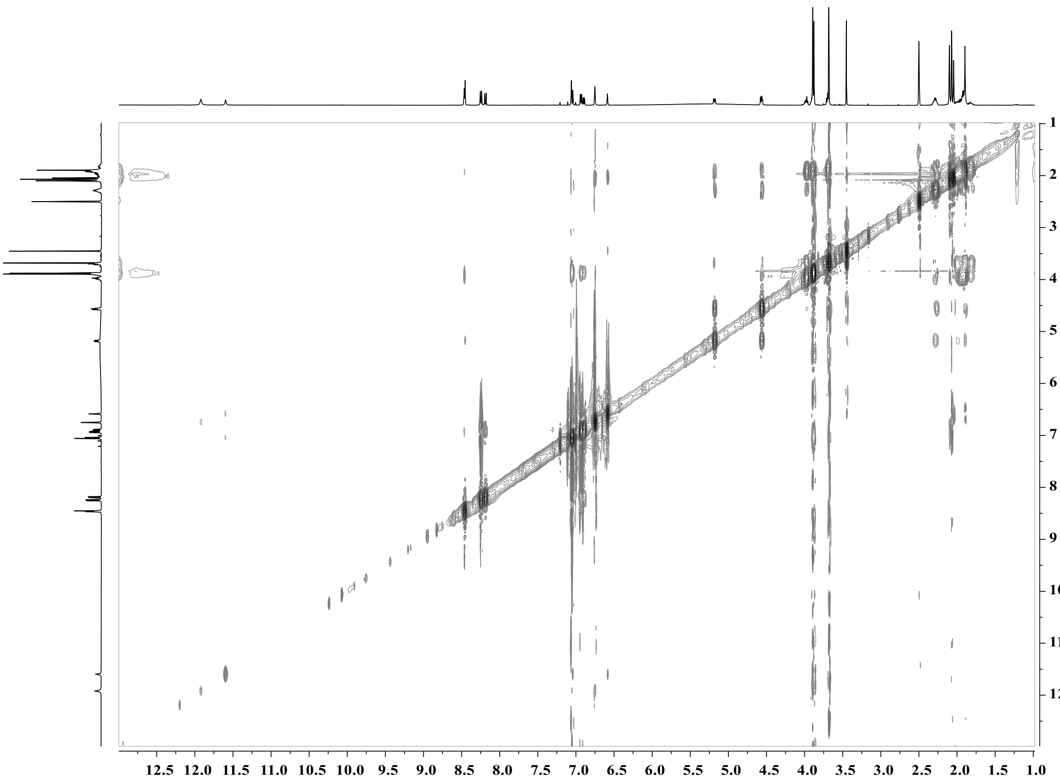

Figure S21. ROESY spectrum of **3** (DMSO-d_6).

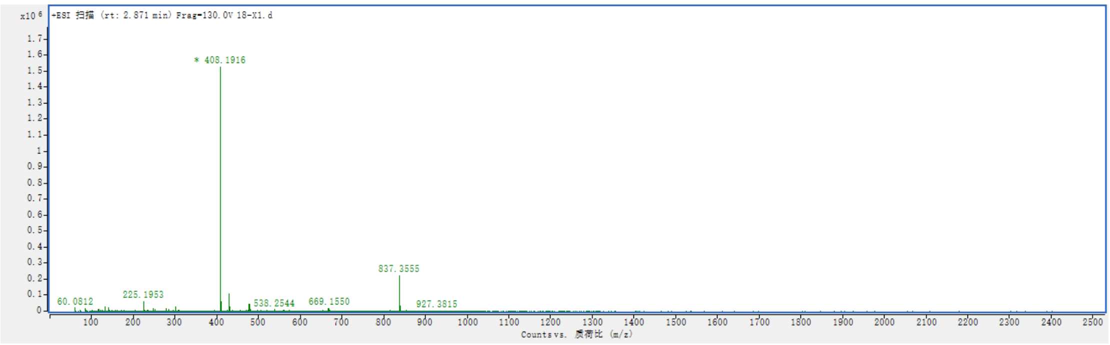

Figure S22. HRESIMS spectrum of **3**.

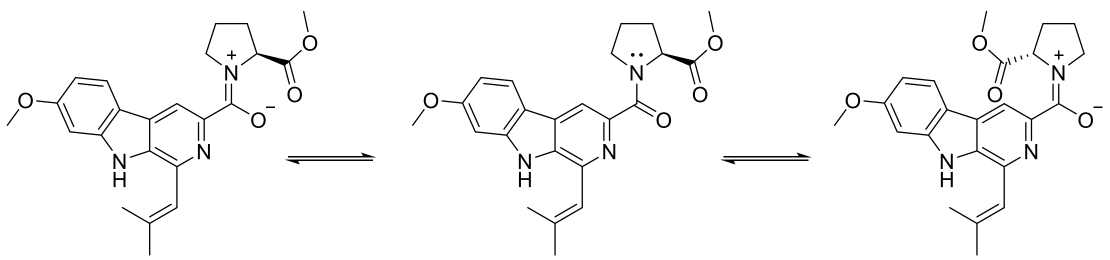

Figure S23. Scheme of the resonance structure of **3** and the chemical equilibrium between **3a** and **3b**.

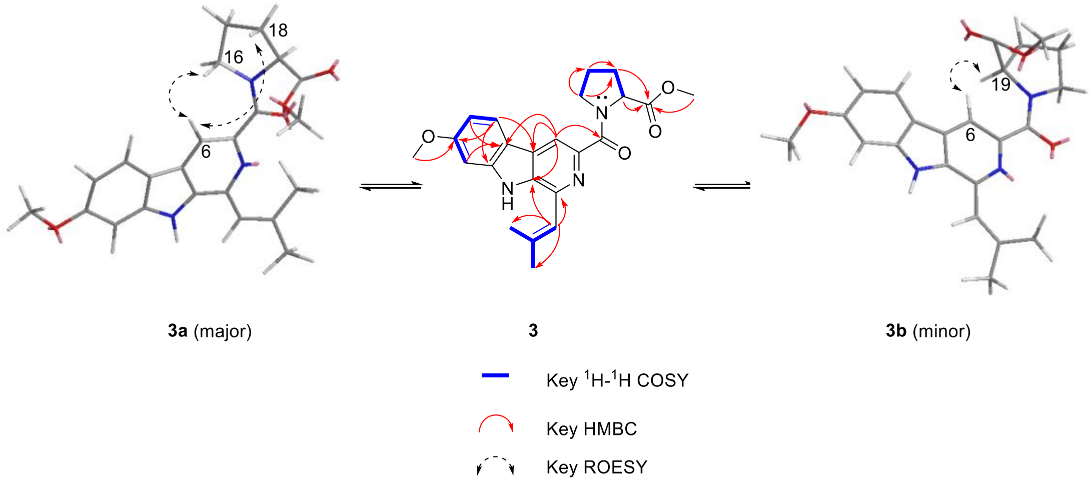

Figure S24. Key ^{1}H-^{1}H COSY, HMBC, and ROESY correlations of **3a** and **3b**.

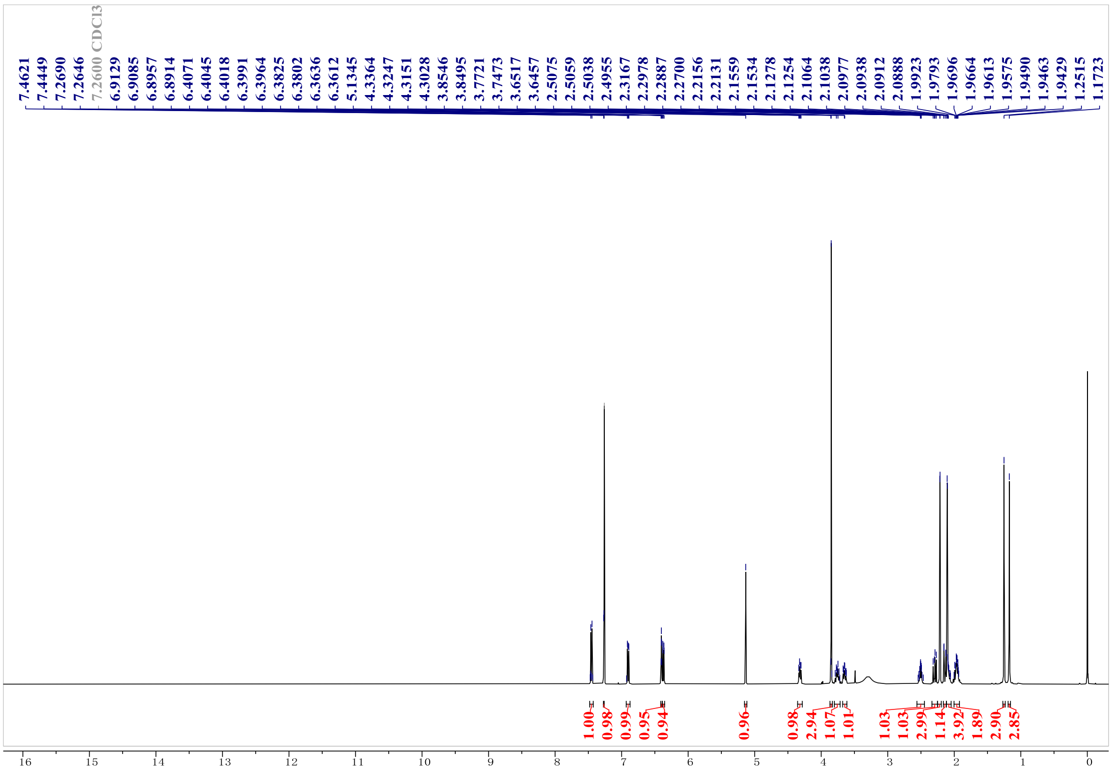

Figure S25. ^{1}H NMR spectrum of **4** ($CDCl_3$-*d*, 500 MHz).

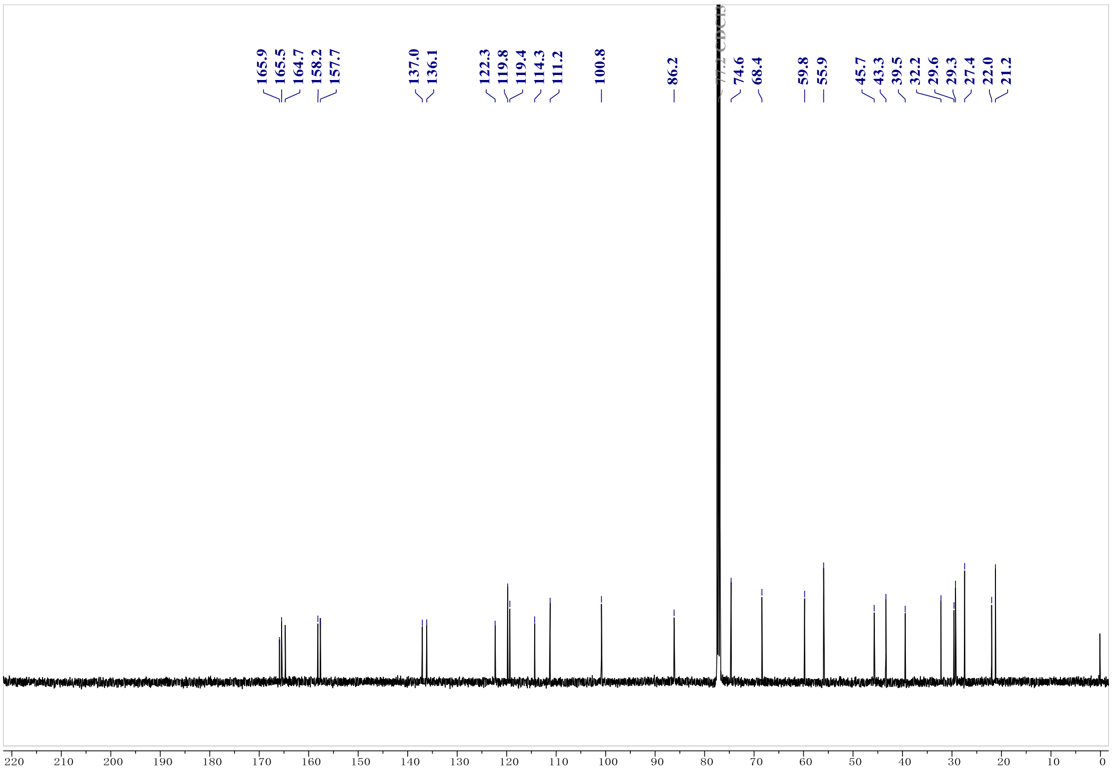

Figure S26. ^{13}C NMR spectrum of **4** ($CDCl_3$-*d*, 125 MHz).

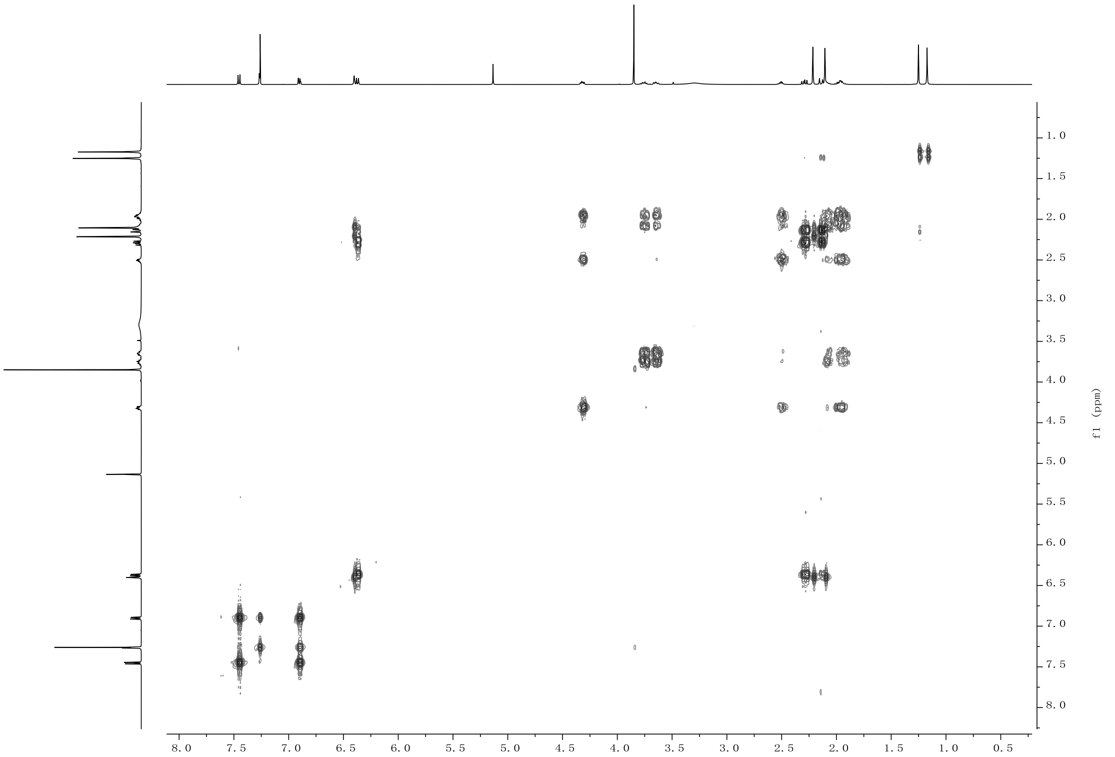

Figure S27. ^{1}H-^{1}H COSY spectrum of **4** ($CDCl_3$-*d*).

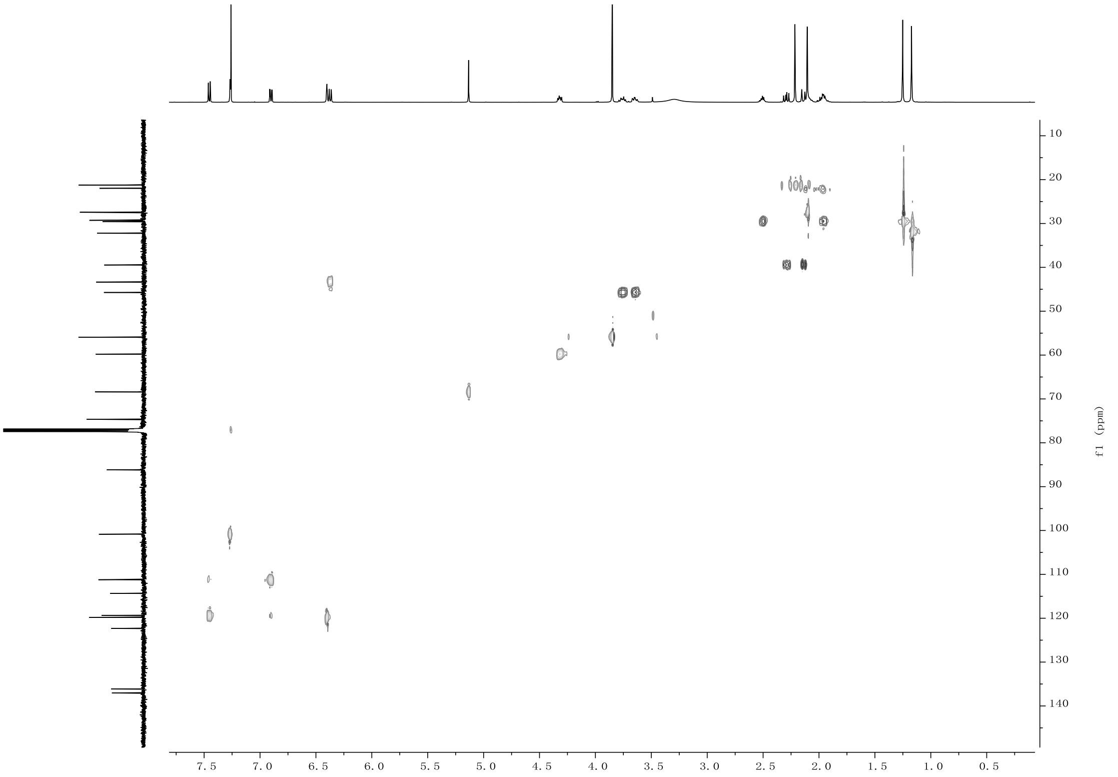

Figure S28. HSQC spectrum of **4** ($CDCl_3$-*d*).

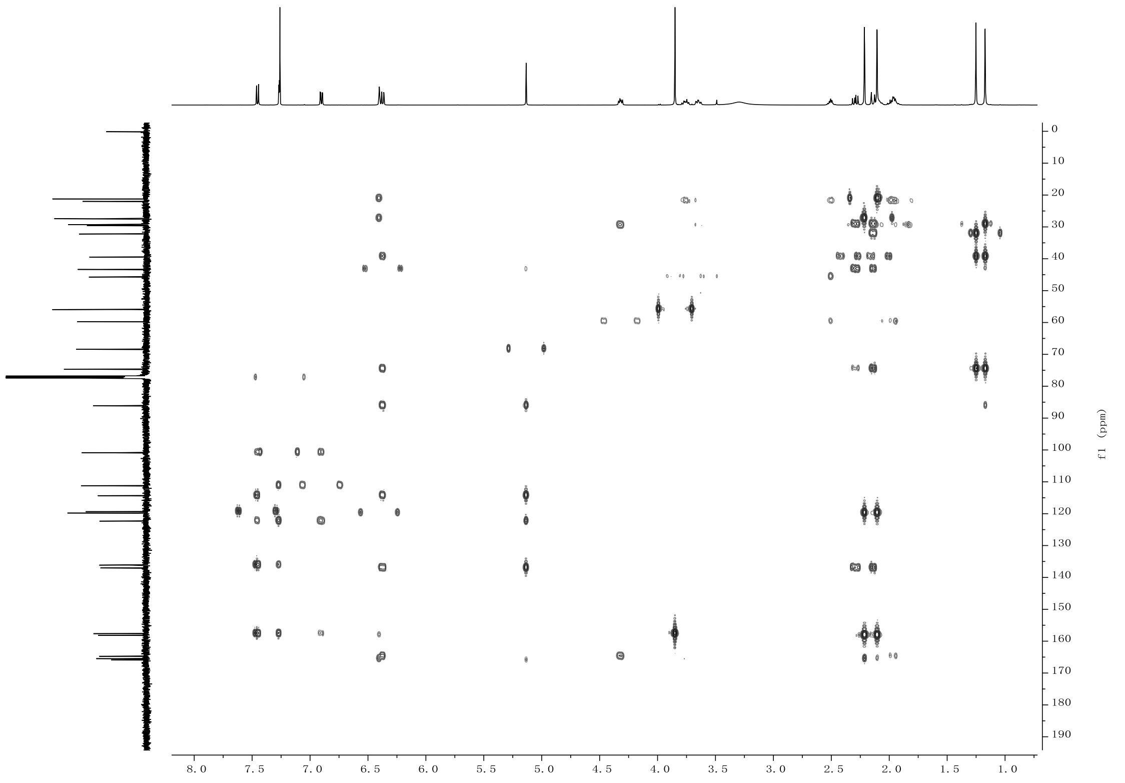

Figure S29. HMBC spectrum of **4** ($CDCl_3$-*d*).

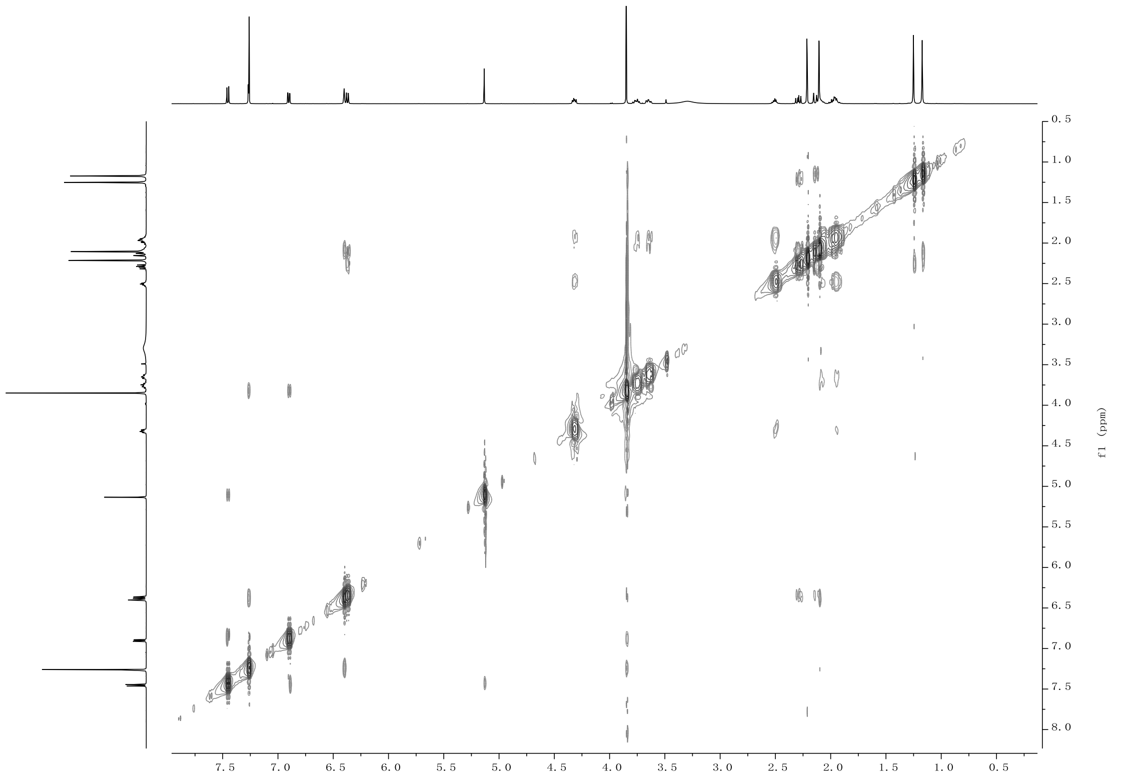

Figure S30. ROESY spectrum of **4** ($CDCl_3$-*d*).

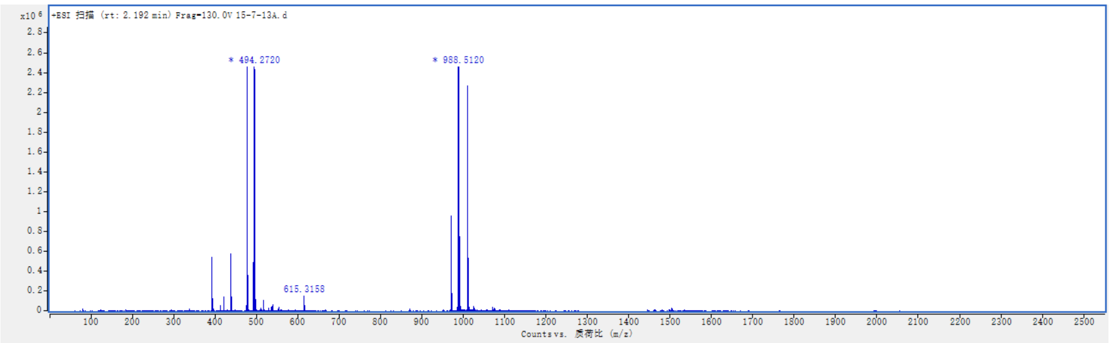

Figure S31. HRESIMS spectrum of **4**.

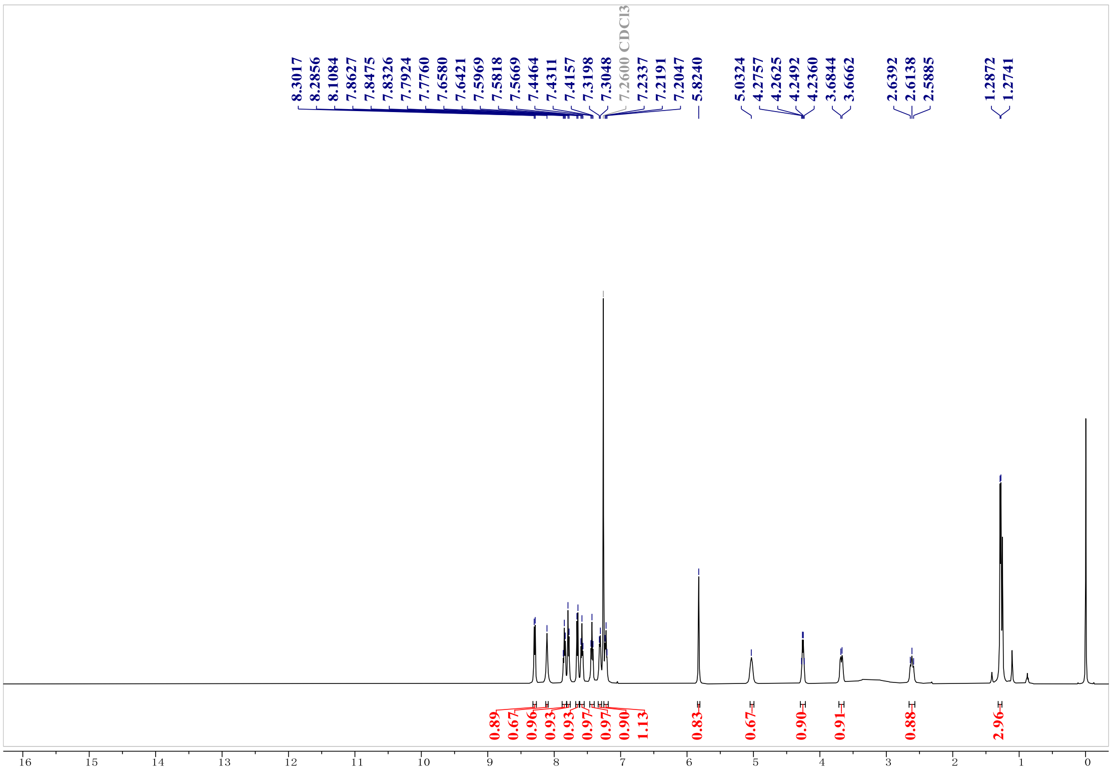

Figure S32. ^{1}H NMR spectrum of **17** ($CDCl_3$-*d*, 500 MHz).

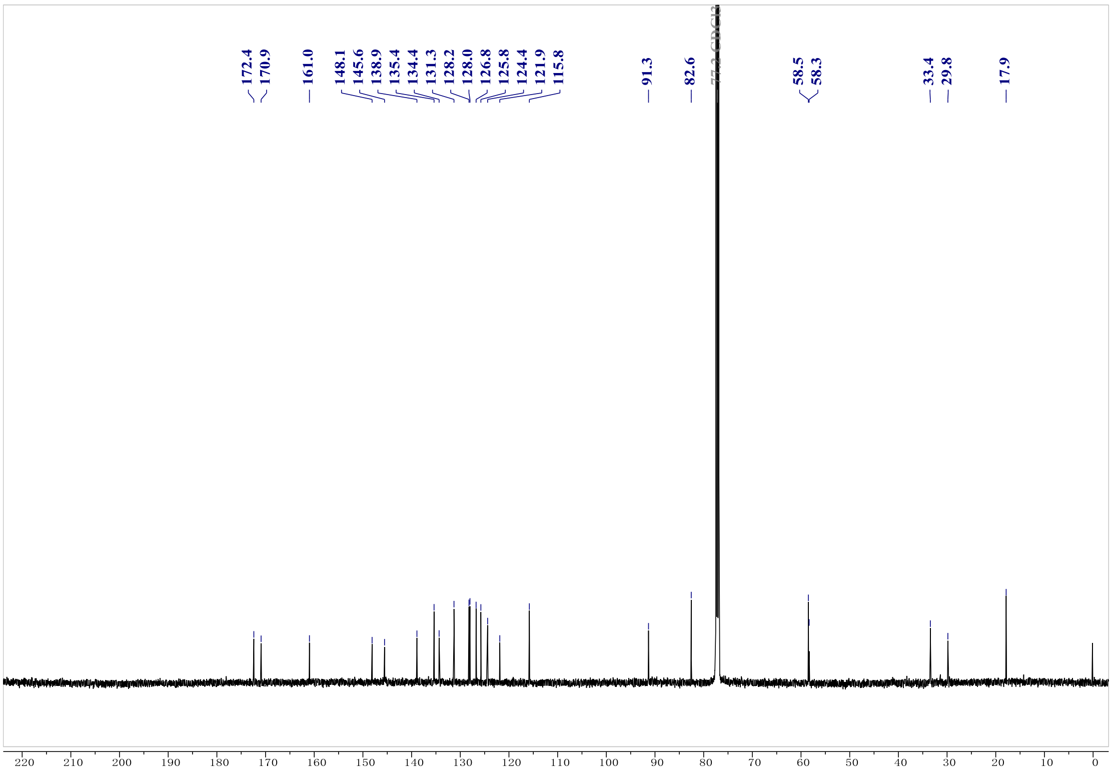

Figure S33. ^{13}C NMR spectrum of **17** ($CDCl_3$-*d*, 125 MHz).

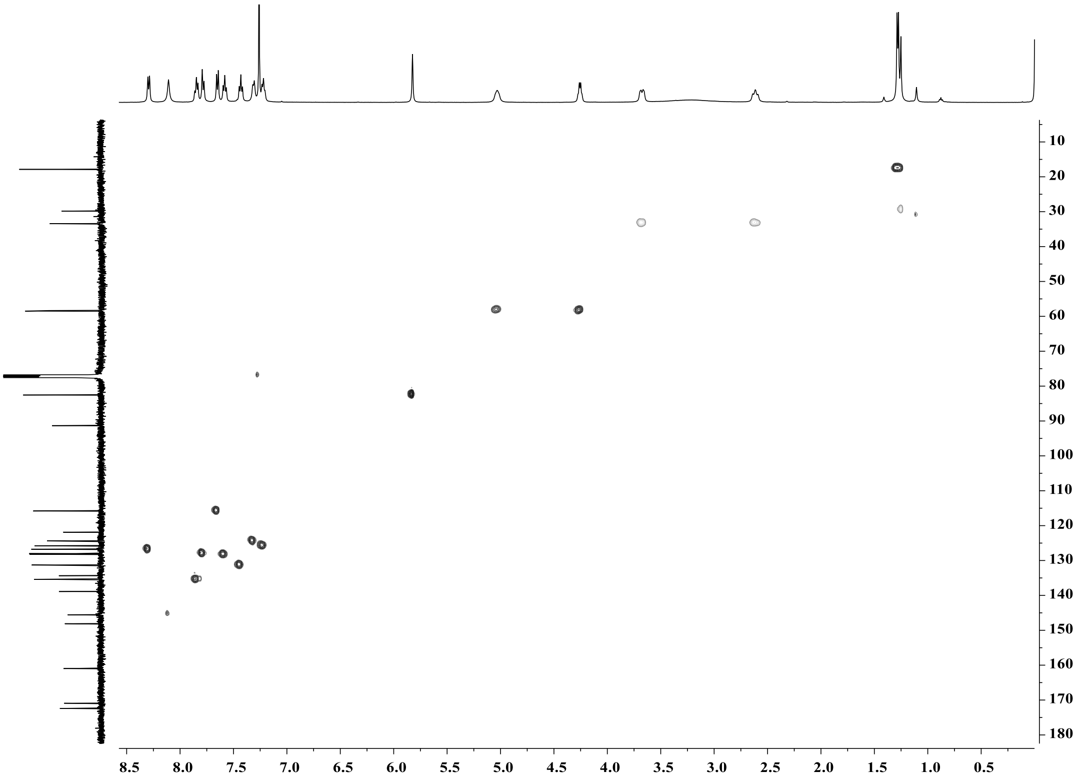

Figure S34. HSQC spectrum of **17** ($CDCl_3$-*d*).

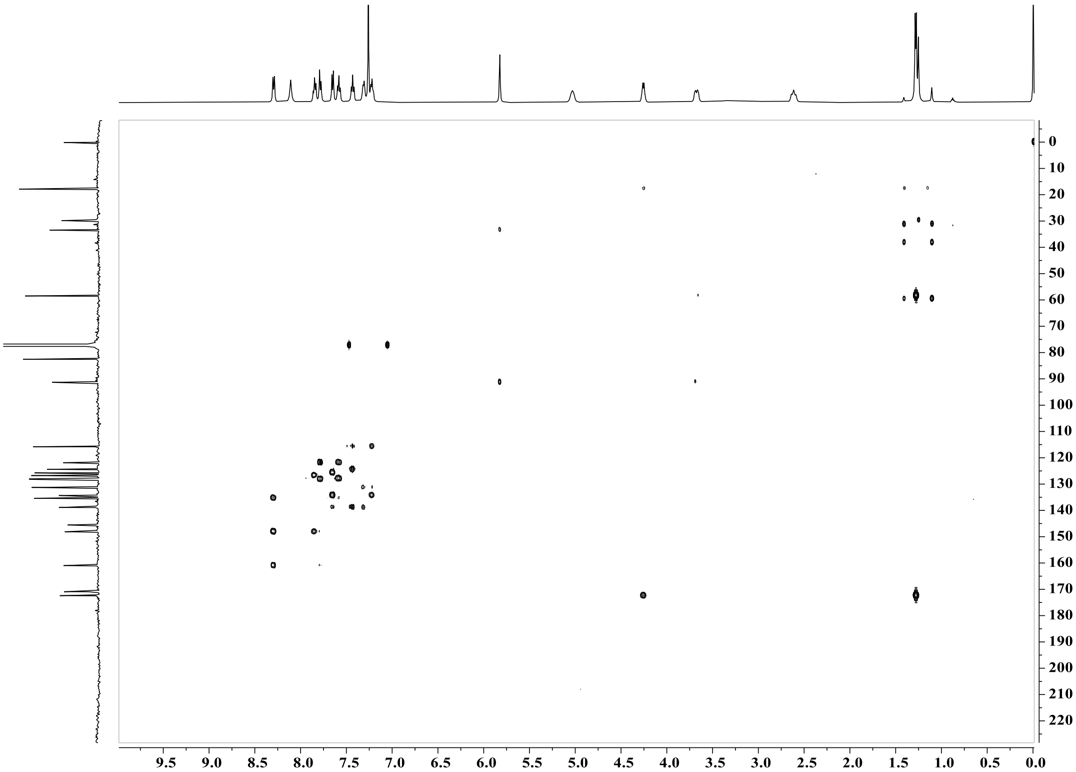

Figure S35. HMBC spectrum of **17** (CDCl3-*d*).

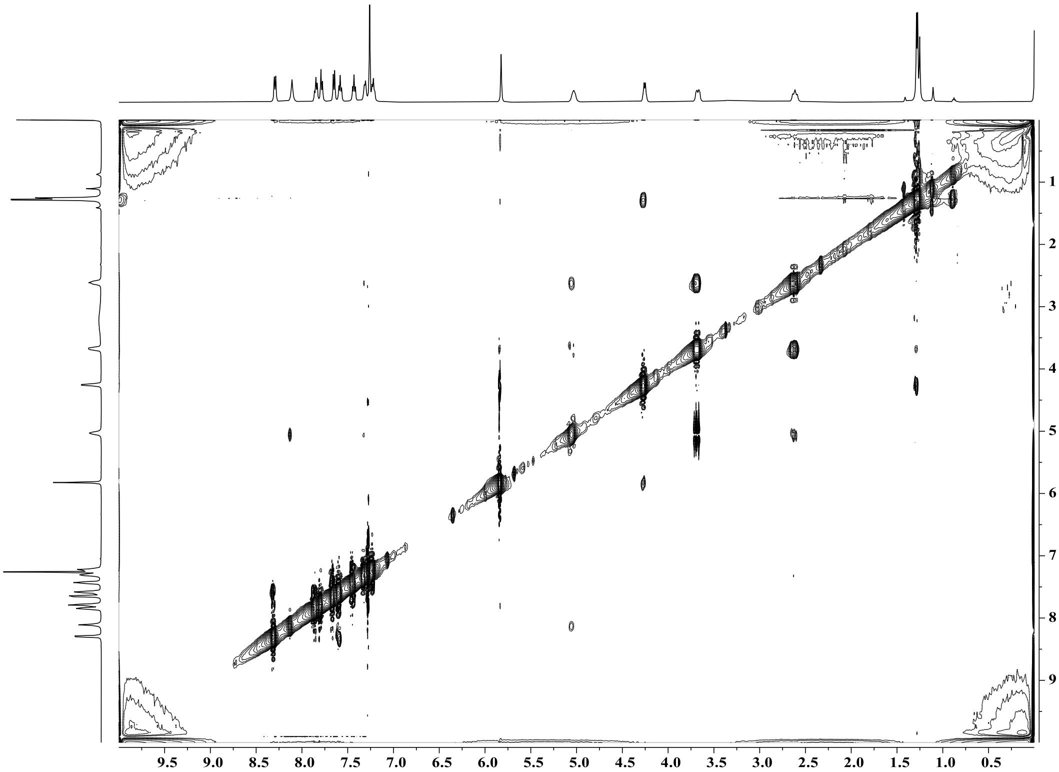

Figure S36. ROESY spectrum of **17** (CDCl3-*d*).

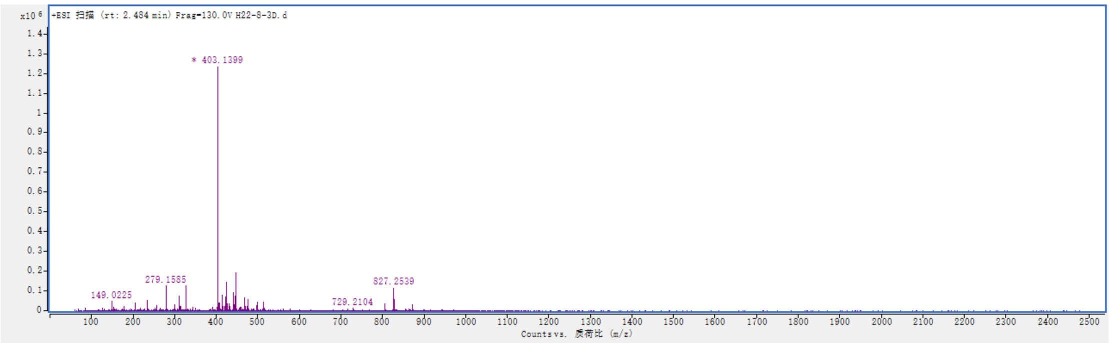

Figure S37. HRESIMS spectrum of **17**.

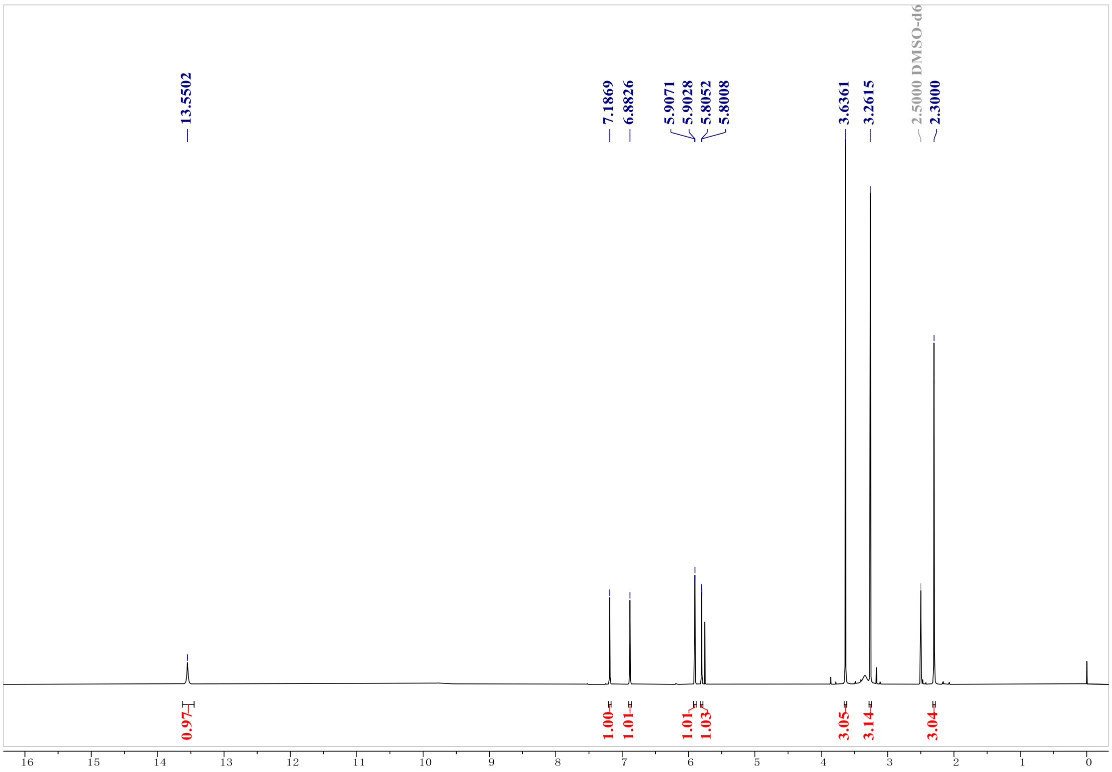

Figure S38. ^{1}H NMR spectrum of **37** (DMSO-d_6, 500 MHz).

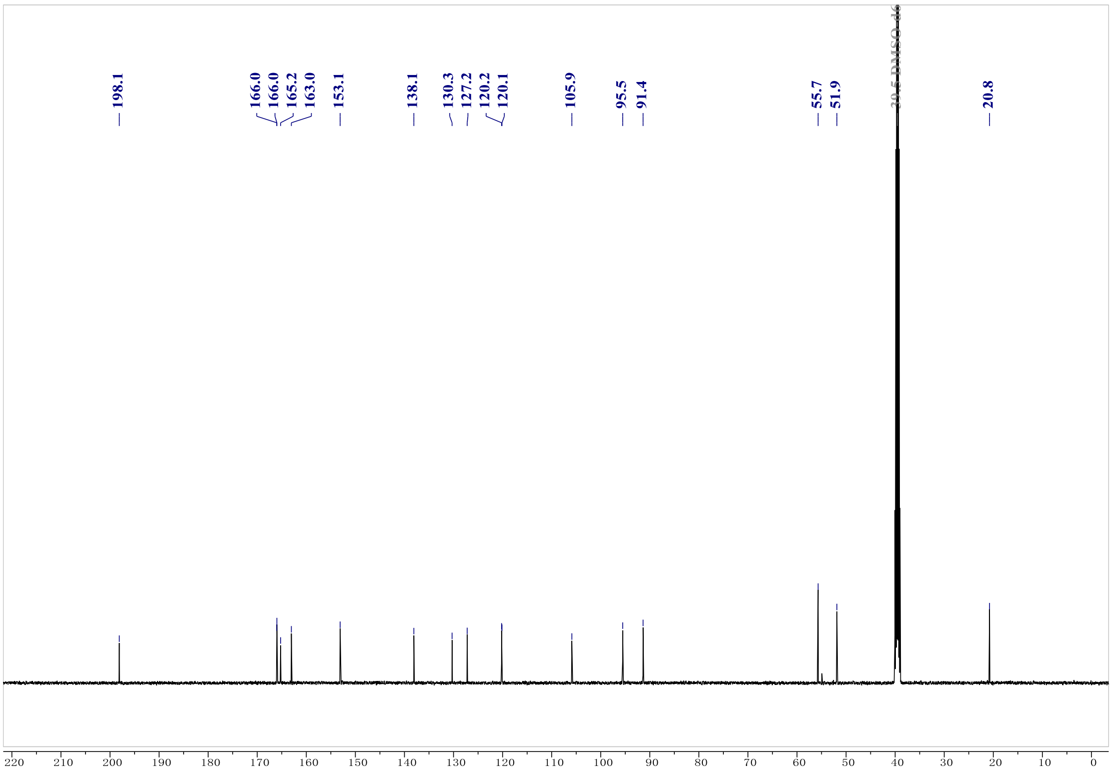

Figure S39. ^{13}C NMR spectrum of **37** (DMSO-d_6, 125 MHz).

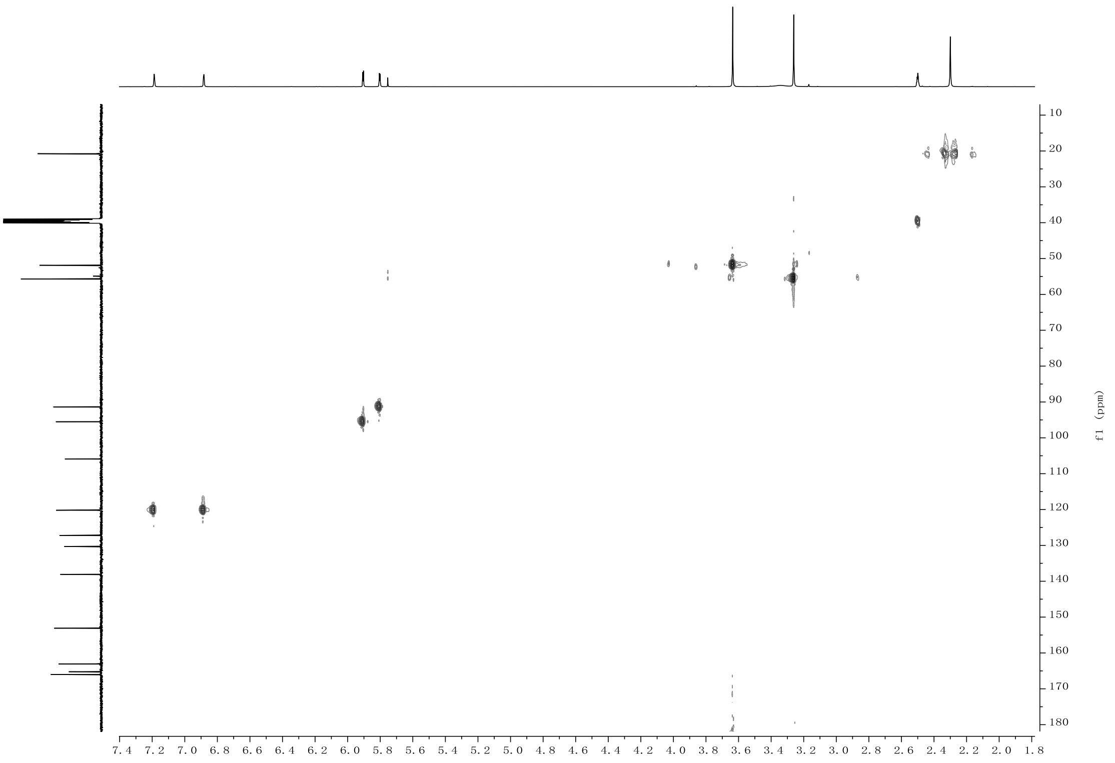

Figure S40. HSQC spectrum of **37** (DMSO-d_6).

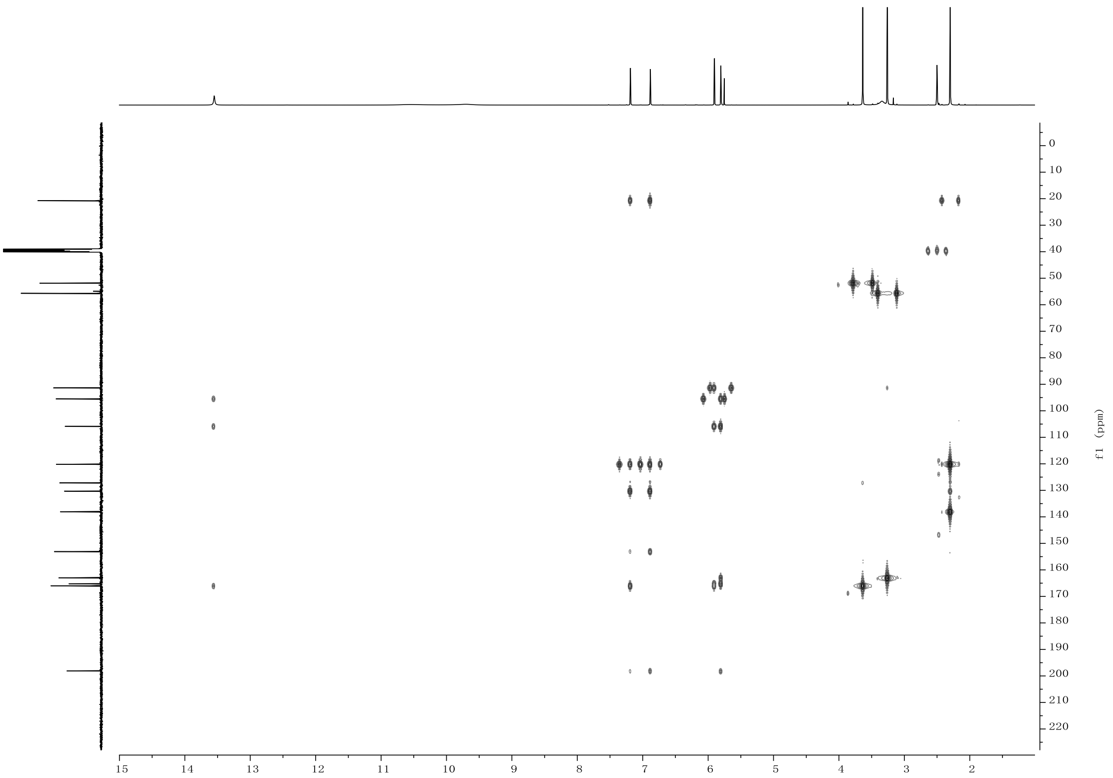

Figure S41. HMBC spectrum of **37** (DMSO-d_6).

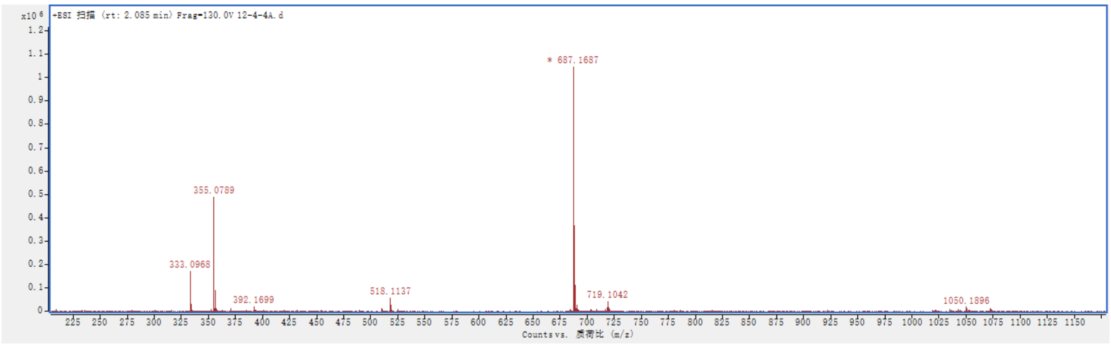

Figure S42. HRESIMS spectrum of **37**.

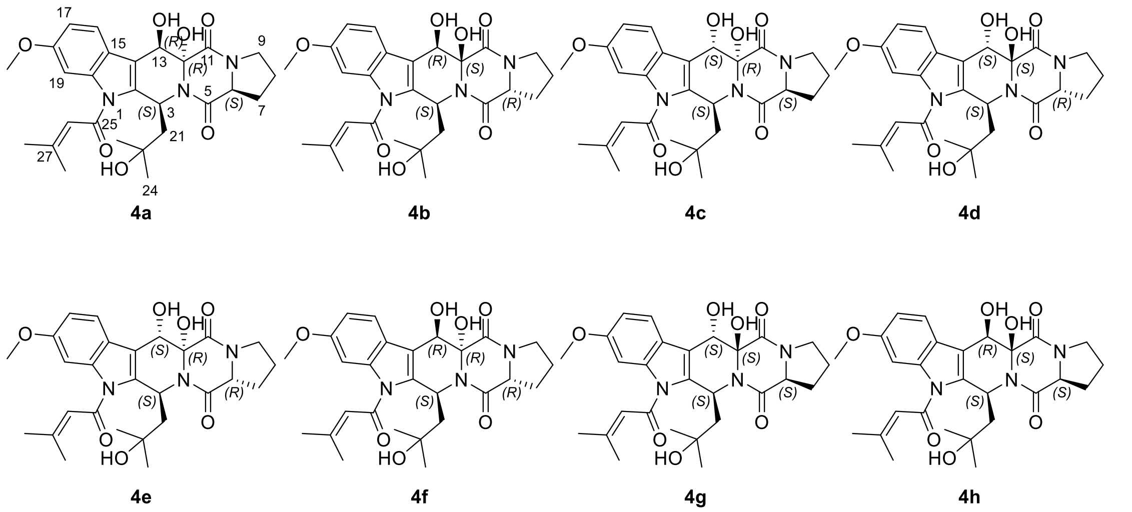

Figure S43. Eight possible stereoisomers of **4** (**4a**–**4h**)

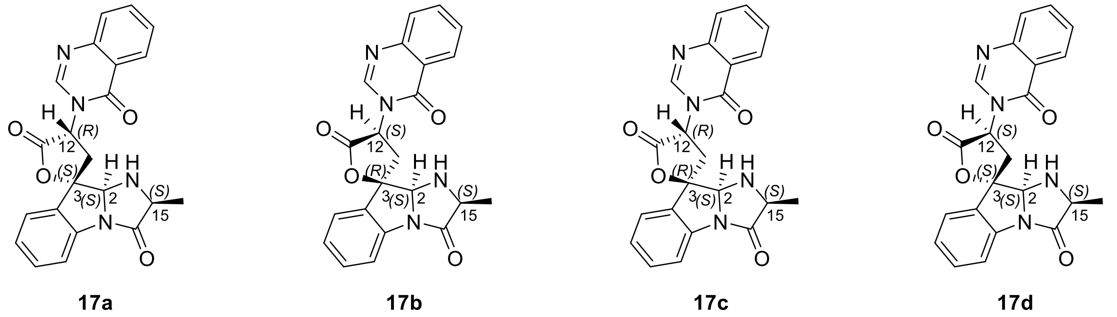

Figure S44. Four possible stereoisomers of **17** (**17a**–**17d**)
